# Electroacoustic activation of natural quartz nanoparticles for the Guareschi–Thorpe synthesis of novel pyridine derivatives

**DOI:** 10.1039/d6ra06595c

**Published:** 2026-10-15

**Authors:** H. A. Morsy, Mohamed G. Assy, Hassan A. El-Sayed, Doaa A. Elsayed

**Affiliations:** a Higher Institution of Engineering & Modern Technology Elmarg Cairo 13774 Egypt; b Department of Chemistry, Faculty of Science, Zagazig University Zagazig 44519 Egypt doaaatef641995@gmail.com doaaatef@zu.edu.eg +20-01207053013; c Department of Medical Laboratory Techniques, Medical Technical College, Al-Farahidi University Baghdad 10021 Iraq; d Anesthesia, Techniques Department, Al-Amal College for Specialized Medical Sciences Karbala Iraq

## Abstract

Natural quartz, the most abundant piezoelectric mineral in the Earth's crust, offers a promising platform for catalysis owing to its intrinsic piezoelectric response, chemical stability, and natural abundance. In this study, natural quartz nanoparticles (NQNs) were prepared from quartz type of (rock crystal) and evaluated as a recyclable catalyst with a proposed piezocatalytic contribution for the Guareschi–Thorpe synthesis of pyridine derivatives. Mechanical stress was applied *via* a newly designed granite resonance chamber that amplified low-frequency acoustic waves (400–500 Hz, function generator) to a sound intensity of 115–117 dB, which is proposed to induce piezoelectric polarization in the NQNs and generate transient surface charges that promote catalytic activation without external heating. Under these electroacoustic conditions, hydroxynicotinonitrile and aminonicotinonitrile derivatives were obtained in excellent isolated yields (92–99%) within markedly short reaction times (10–25 min) at ambient temperature. Comparative experiments performed under three conditions (i) NQNs combined with acoustic stimulation, (ii) acoustic stimulation alone, and (iii) conventional stirring without either NQNs or acoustic activation showed that NQNs markedly accelerate the acoustically driven reaction: acoustic irradiation alone required four- to twelve-fold longer reaction times with lower yields, while conventional stirring afforded no detectable product even after prolonged standing. Compared with a previously reported thermal protocol requiring 80 °C for up to 300 min, the present method reduced reaction time by more than 90% while maintaining comparable or superior yields. The catalyst retained its activity over six consecutive cycles with only a marginal yield decrease (98% to 95%), confirming its robustness and recyclability. These findings identify natural quartz nanoparticles as an effective, recyclable catalyst for acoustically driven multicomponent heterocyclic synthesis, with a lower estimated energy input than the thermal benchmark; the piezoelectric contribution is proposed and remains to be proven mechanistically.

## Abstract 2

Natural quartz nanoparticles act as an efficient piezoelectric catalyst for the Guareschi–Thorpe synthesis of novel pyridine derivatives under acoustic activation, providing a simple and recyclable approach to heterocyclic compounds.

## Graphical abstract

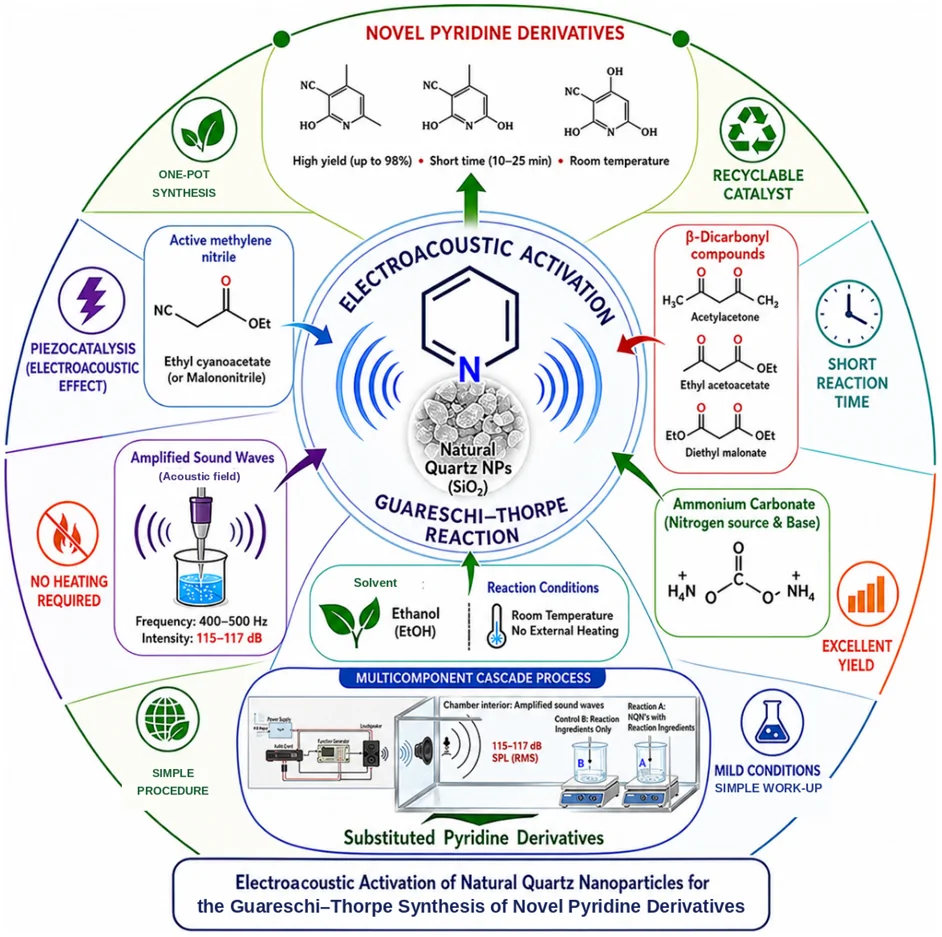

## Introduction

Nitrogen-containing heterocyclic compounds constitute one of the most important structural motifs in modern organic chemistry owing to their broad distribution in pharmaceuticals, agrochemicals, natural products, functional materials, and coordination chemistry. Among these heterocyclic systems, pyridine derivatives represent one of the most privileged scaffolds because of their unique electronic properties, synthetic versatility, and remarkable biological activities. The incorporation of a pyridine ring frequently enhances aqueous solubility, metabolic stability, and receptor-binding affinity, making pyridine-containing molecules indispensable building blocks in medicinal chemistry and drug discovery.^1–3^ Pyridine derivatives are present in numerous clinically approved drugs and naturally occurring bioactive compounds. Representative examples include the anticancer alkaloid Camptothecin, whose pyridone-containing framework is essential for topoisomerase I inhibition, the anti-HIV drug Doravirine, the cyclin-dependent kinase inhibitor Palbociclib used in breast cancer therapy, and the naturally occurring alkaloid Huperzine A, which exhibits potent acetylcholinesterase inhibitory activity for the treatment of Alzheimer's disease. In addition, pyridine-based scaffolds have been incorporated into numerous antimicrobial, antiviral, antitumor, anti-inflammatory, antioxidant, and enzyme inhibitory agents, highlighting their exceptional pharmacological importance,^4–8^Fig. 1.

**Fig. 1:**
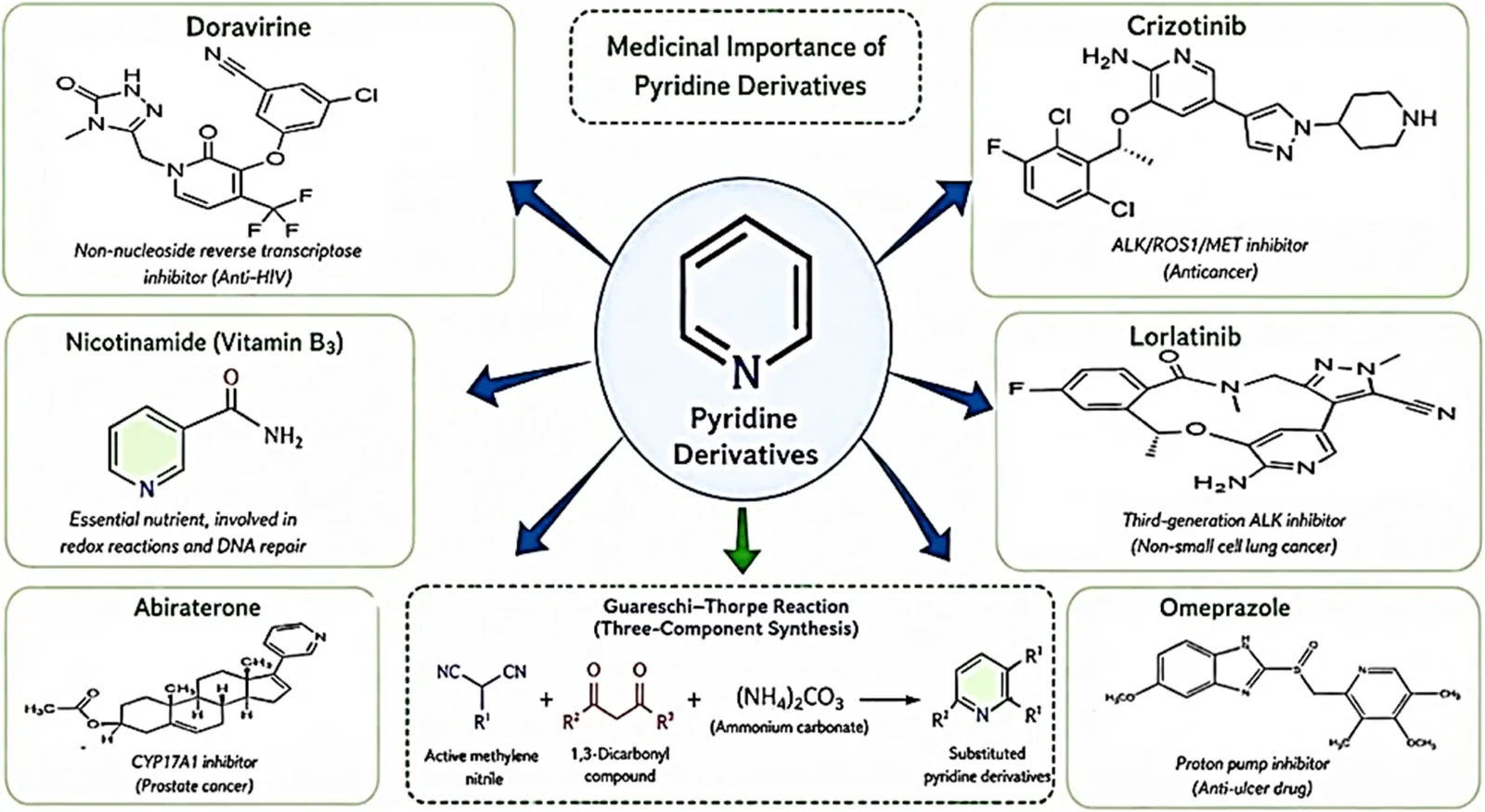
Pharmaceutical importance of pyridine derivatives.

Beyond pharmaceutical applications, pyridine derivatives have attracted increasing attention in materials science owing to their excellent coordination ability toward transition metals and their favorable electronic characteristics. They have found extensive applications as ligands in metal complexes, fluorescent probes, corrosion inhibitors, organic semiconductors, dyes, catalysts, sensors, and supramolecular assemblies. Consequently, the development of efficient synthetic methodologies for structurally diverse pyridine derivatives remains an active area of research.^9–11^

Among the numerous synthetic approaches available for pyridine construction, the Guareschi–Thorpe reaction remains one of the most practical multicomponent strategies because it enables the direct assembly of highly substituted pyridine frameworks from readily available starting materials in a single synthetic operation. Conventionally, this transformation involves the condensation of an active methylene nitrile, a β-dicarbonyl compound, and ammonia or an ammonium salt to generate polysubstituted pyridine derivatives through sequential Knoevenagel condensation, Michael addition, cyclization, and aromatization steps.^12–14^

Despite its synthetic efficiency, many classical Guareschi–Thorpe procedures rely on prolonged refluxing, hazardous organic solvents, strong bases, or harsh reaction conditions, which increase energy consumption and reduce sustainability. Therefore, considerable efforts have recently been devoted to redesigning this valuable transformation according to the principles of green chemistry. A notable advance was reported through the Guareschi–Thorpe synthesis of pyridines in green buffer and pH-controlled aqueous medium using ammonium carbonate, where ammonium carbonate acted simultaneously as a nitrogen source and buffering reagent, allowing efficient pyridine synthesis under aqueous conditions with simplified work-up and reduced chemical waste.^15^

Catalysis represents one of the twelve fundamental principles of green chemistry because catalysts accelerate chemical reactions without being consumed, thereby reducing energy requirements, minimizing reagent consumption,^16–18^ improving reaction selectivity, and suppressing undesired side reactions. In particular, nanocatalysts have emerged as highly efficient catalytic systems owing to their exceptionally large surface-area-to-volume ratio and high density of accessible active sites.^19^ Their catalytic performance strongly depends on nanoparticle size, morphology, and composition, enabling significant improvements in reaction rates, selectivity, and product yields while reducing environmental impact.^20^

Natural minerals have recently attracted increasing attention as heterogeneous catalysts for organic synthesis. Clay minerals, zeolites, silicates, carbonates, phosphates, and related naturally occurring materials have successfully promoted numerous organic transformations under mild reaction conditions, demonstrating that abundant natural minerals can serve as inexpensive, recyclable, catalytic materials.^21,22^

Among naturally occurring minerals, quartz (α-SiO_2_) – (rock crystal type) is particularly attractive because of its excellent chemical stability, mechanical robustness, natural abundance, and intrinsic piezoelectric properties. Unlike centrosymmetric materials, the non-centrosymmetric crystal structure of quartz undergoes deformation when subjected to external mechanical stress, generating transient electrical polarization through the direct piezoelectric effect. This phenomenon enables quartz to convert mechanical energy into electrical energy, providing an electroacoustic response that can facilitate charge separation, activate reactants, and accelerate catalytic processes.^23–28^

It should be emphasized, however, that respirable crystalline silica, including quartz nanoparticles, is a recognized occupational inhalation hazard: chronic exposure is associated with pulmonary inflammation and fibrosis (silicosis), oxidative stress, and an increased risk of lung cancer, and nanoparticulate forms may show enhanced toxicity because of their high surface reactivity. Natural quartz nanoparticles are therefore not regarded here as benign; their use requires dust control and personal protective equipment, and the handling and spill-management precautions are summarized in the Experimental section and in the SI.

Piezoelectric catalysis (piezocatalysis) has recently emerged as an attractive branch of catalysis, in which mechanical energy is directly converted into chemical activity through piezoelectric materials. Unlike photocatalysis, which relies on electromagnetic radiation, or pyrocatalysis, which is driven by thermal fluctuations, piezocatalysis utilizes mechanical vibrations to generate surface charges capable of promoting chemical transformations. Various synthetic piezoelectric materials, including BaTiO_3_, LiNbO_3_, NaNbO_3_, and ZnO,^21,29,30^ have demonstrated remarkable catalytic performance under ultrasonic or mechanical excitation. Nevertheless, their relatively high cost, complicated preparation, and limited sustainability have encouraged the exploration of naturally occurring piezoelectric materials as more abundant and lower-cost alternatives.^29,31,32^

Ultrasound-assisted organic synthesis (UAOS) has emerged as an attractive green technology capable of significantly accelerating chemical transformations while reducing reaction times, solvent consumption, and energy requirements. The enhanced reaction efficiency originates from acoustic cavitation, which generates localized transient hot spots characterized by extremely high temperatures and pressures that facilitate bond formation without increasing the bulk reaction temperature.^33–36^

Inspired by these advances, the present work introduces an electroacoustic strategy based on naturally occurring quartz nanoparticles for the Guareschi–Thorpe synthesis of novel pyridine derivatives. Under amplified sound-wave irradiation from a heavy (granite resonance box), the piezoelectric response of natural quartz nanoparticles is proposed to generate transient surface charges that may promote reactant activation and accelerate multicomponent cyclization under ambient conditions. The combination of recyclable natural quartz nanoparticles, ammonium carbonate as a convenient nitrogen source, and ethanol as a comparatively benign solvent provides a rapid and high-yielding protocol without external heating, thereby expanding the application of naturally occurring piezoelectric minerals in heterocyclic synthesis.

## Results and discussions

### Field emission scanning electron microscopy (FESEM) analysis of natural quartz nanoparticles

The surface morphology, particle size, and microstructural characteristics of the natural quartz nanoparticles (NQNPs) were investigated using field emission scanning electron microscopy (FESEM). Representative FESEM micrographs are presented in Fig. 2 and 3. The FESEM images reveal that the NQNPs possess an irregular, rough, and non-smooth surface morphology with good particle dispersion and minimal agglomeration. Such morphological characteristics provide a high density of accessible active sites, which are expected to enhance the catalytic performance of the material by increasing the effective surface area available for reactant adsorption.

**Fig. 2:**
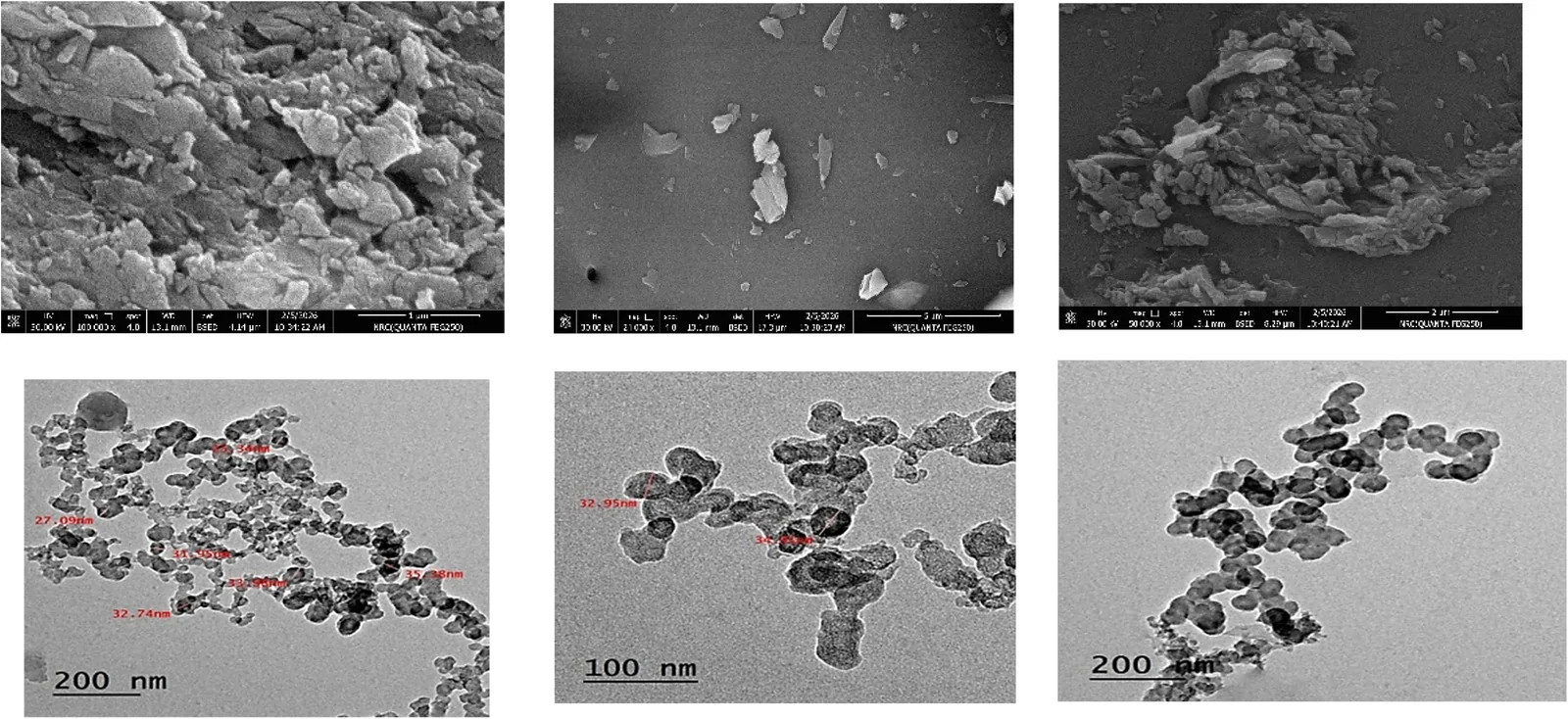
FESEM images of the natural quartz nanoparticle.

**Fig. 3:**
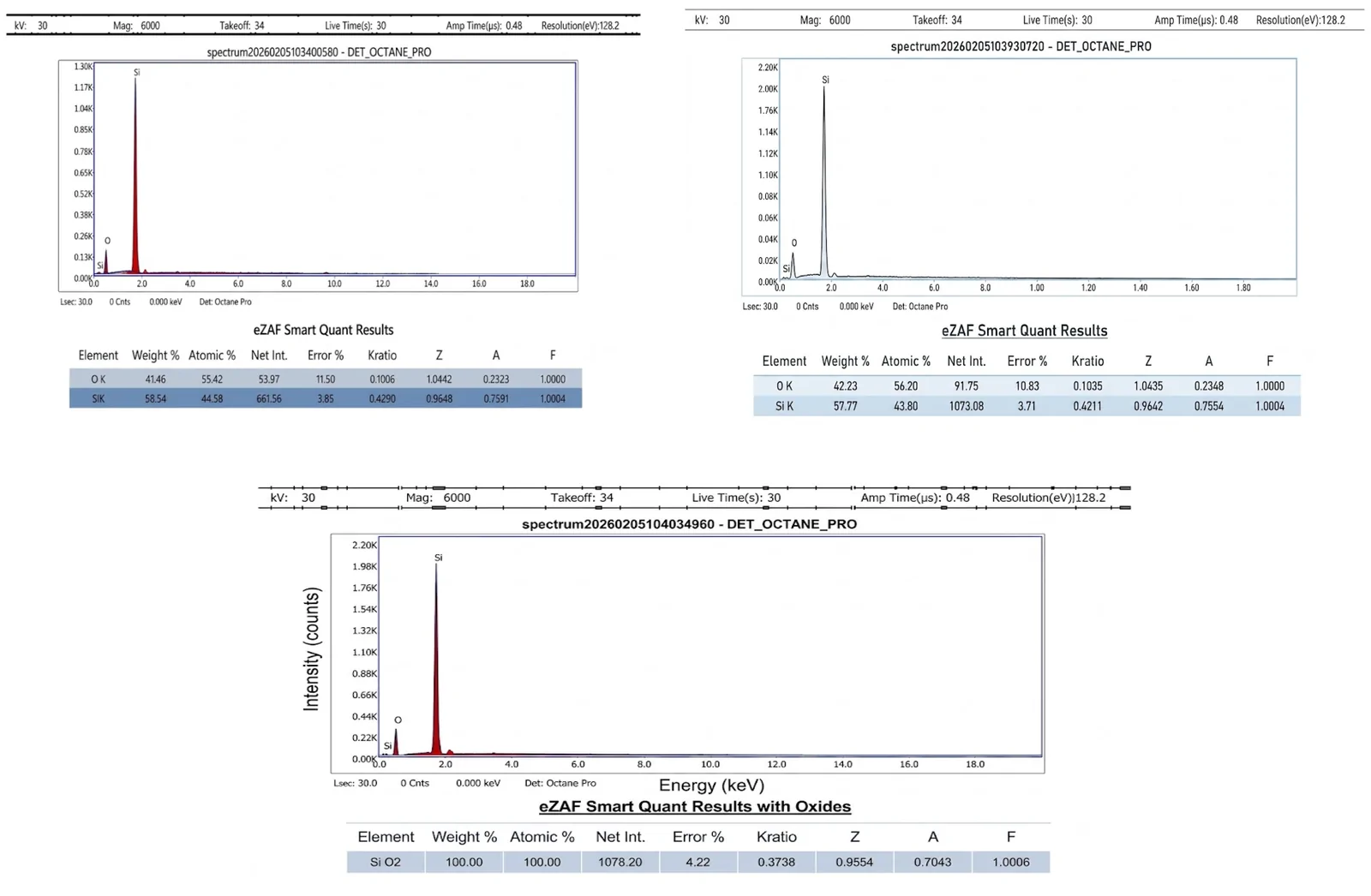
EDX – spectrum of natural quartz catalyst sample.

Furthermore, the nanoparticles exhibit a relatively uniform size distribution with an average particle diameter ranging from 25 to 35 nm. The nanoscale dimensions, combined with the rough surface texture, contribute significantly to the high catalytic activity of the natural quartz nanoparticles by facilitating efficient mass transfer and increasing the number of exposed active sites. These observations confirm that the prepared NQNPs possess the desirable structural features required for efficient heterogeneous catalysis.

### X-ray diffraction (XRD) analysis of natural quartz nanoparticles

The crystalline structure and phase purity of the prepared natural quartz nanoparticles (NQNPs) were investigated by X-ray diffraction (XRD). The diffraction pattern, shown in Fig. 4, exhibited a series of sharp and well-defined reflections at 2*θ* values of approximately 20.86°, 26.64°, 36.55°, 39.47°, 40.30°, 42.46°, 45.80°, 50.15°, 50.63°, 54.88°, 55.34°, and 57.24°. These diffraction peaks were indexed to the crystallographic planes (100), (101), (110), (102), (111), (200), (201), (112), (003), (202), (103), and (210), respectively.

**Fig. 4:**
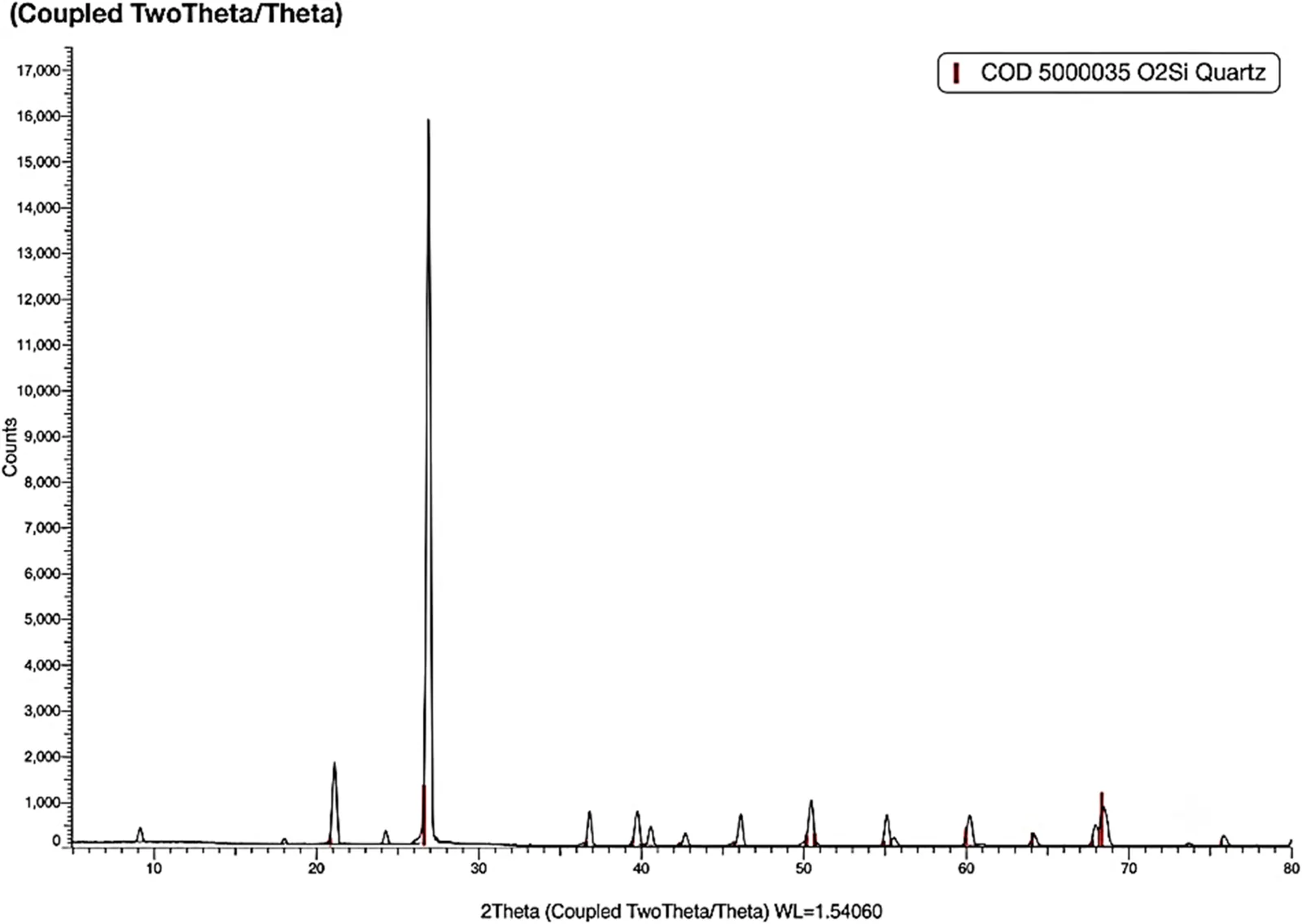
XRD pattern of natural quartz NPs.

The observed diffraction pattern is characteristic of crystalline α-quartz (SiO_2_) and confirms the high crystallinity and phase purity of the prepared nanoparticles. The diffraction peaks are in excellent agreement with the standard crystallographic data reported in the Crystallography Open Database (COD no. 5000035), confirming the formation of a single crystalline quartz phase without detectable impurity phases.

Furthermore, the indexed reflections are consistent with the trigonal crystal structure of α-quartz belonging to the space group *P*3_2_21 (no. 154) (or its enantiomorphic counterpart *P*3_1_21, depending on the reference database). The sharp diffraction peaks and the absence of additional reflections indicate that the quartz nanoparticles possess a highly ordered crystalline framework, which is expected to contribute to their excellent catalytic performance by providing structurally stable and well-defined surface active sites.

The obtained XRD pattern is in excellent agreement with the standard reference pattern of α-quartz (JCPDS Card no. 01-085-0930), confirming the formation of highly crystalline quartz nanoparticles. The most intense diffraction peak observed at 2*θ* = 26.64°, corresponding to the (101) crystallographic plane, is characteristic of crystalline quartz and indicates the high crystallinity of the prepared nanoparticles. The refined lattice parameter was determined to be *a* = 4.912 Å, which is in close agreement with the reported crystallographic data for α-quartz possessing a trigonal crystal structure (hexagonal setting). These findings further verify the successful formation of phase-pure quartz nanoparticles with excellent crystallinity and structural stability, making them suitable as heterogeneous catalysts.^37^

### Pyridines derivative s cyclization &structural analysis of the synthesized compounds

The synthesis of hydroxynicotinonitrile derivatives (1–3) was successfully achieved through a one-pot three-component Guareschi–Thorpe condensation involving ethyl cyanoacetate, ammonium carbonate, and different 1,3-dicarbonyl compounds in the presence of natural quartz nanoparticles (NQNPs) under amplified acoustic irradiation (115–117 dB) at room temperature Table 1. This protocol afforded the desired pyridine derivatives in excellent isolated yields (97–99%) within remarkably short reaction times (10–25 min).

**Table 1:** Comparative study of reaction time and yield for the synthesis of pyridine derivatives (**1**, **2**, **3**) under amplified acoustic activation, conventional stirring, and the reported method

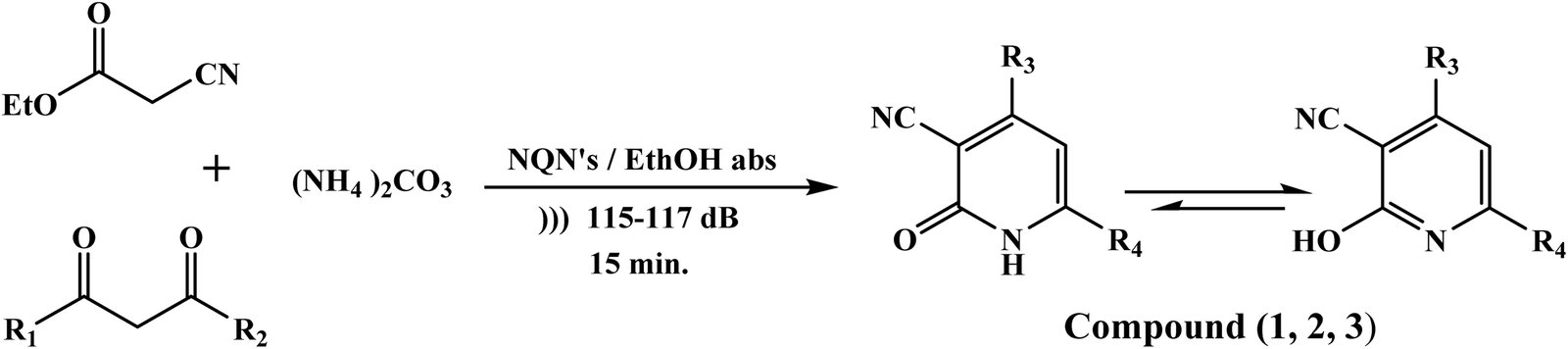																			
Entry	*R*_1_	*R*_2_	Product	Time (min)				Yield%				Catalyst				Reaction conditions			
				This study method			Ref. no.	This study method			Ref. no.	This study method			Ref. no.	This study method			Ref. no.
				*A*	*B*	*C* over night	15	*A*	*B*	*C*	15	*A*	*B*	*C*	15	*A*	*B*	*C*	15
1	CH_3_	CH_3_	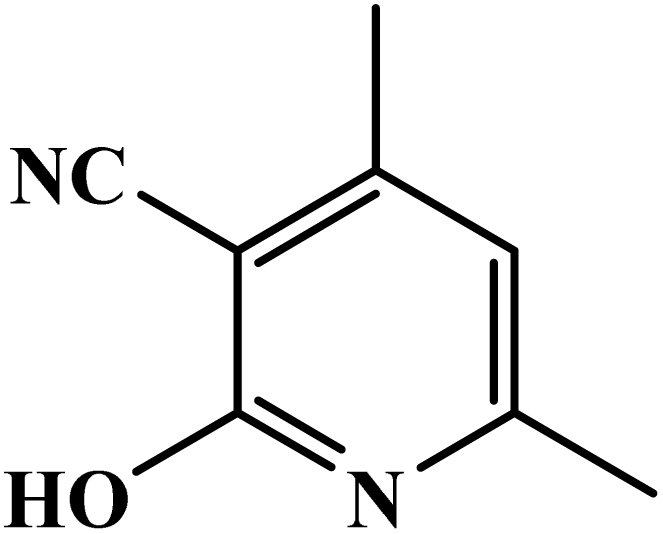	15	60	NR	300	98	60	0	95	NQN's	—	—	—	Amplified sound waves/NQN's	Amplified sound waves	Standing only at RT	Stirring 80 °C
2	CH_3_	OEt	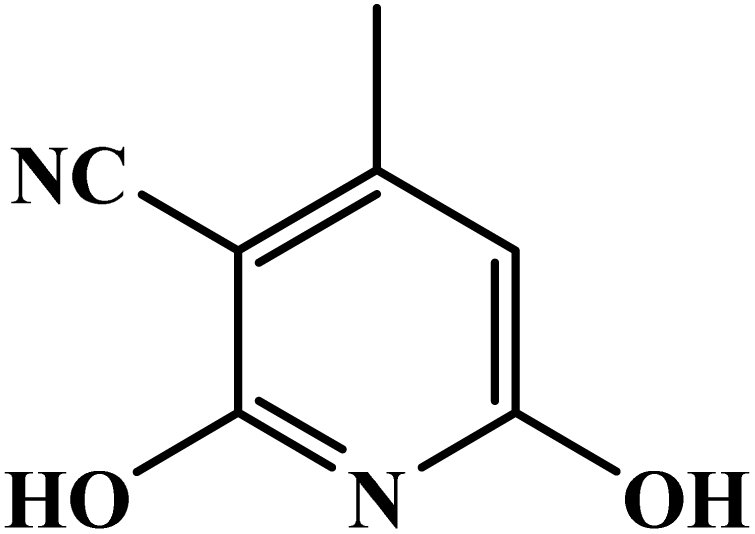	15	60	NR	240	97	66	0	98	NQN's	—	—	—	Amplified sound waves/NQN's	Amplified sound waves	Standing only at RT	Stirring 80 °C
3	OEt	OEt	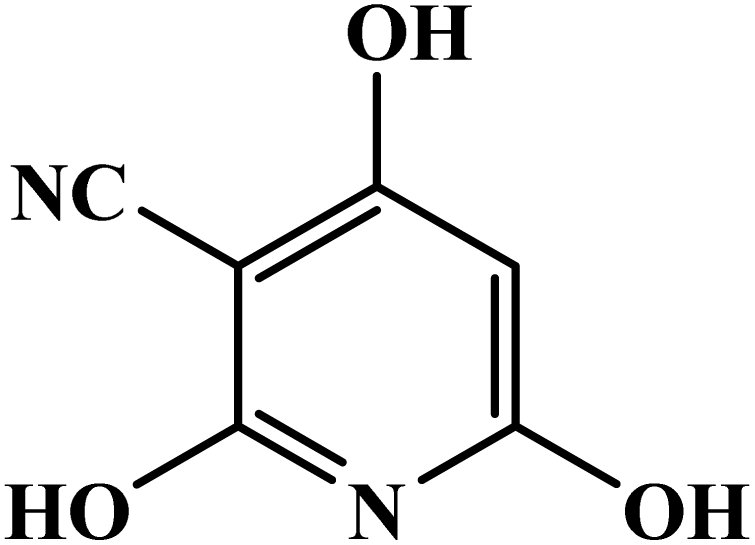	25	180	NR	—	99	63	0	—	NQN's	—	—	—	Amplified sound waves/NQN's	Amplified sound waves	Standing only at RT	Stirring 80 °C

The reaction represents an alternative to conventional heating because no external thermal energy was required, and product isolation involved only simple filtration followed by recrystallization from ethanol. Compared with previously reported procedures based on aqueous heating or prolonged stirring, the present methodology significantly decreases reaction time while maintaining or slightly improving product yields. The observed acceleration may be attributed to the combined action of the acoustic field (enhanced mixing and mass transfer) and the catalytic surface of natural quartz nanoparticles, which may increase the collision frequency between reactants and stabilize key reaction intermediates; the relative contribution of these effects, including a possible piezoelectric effect, is discussed below.

To evaluate the contribution of each activation mode, three experimental protocols were examined: Method A: amplified sound waves + NQNPs, Method B: amplified sound waves only, Method C: standing at room temperature. The obtained results are summarized in Table 1 and Fig. 5.

**Fig. 5:**
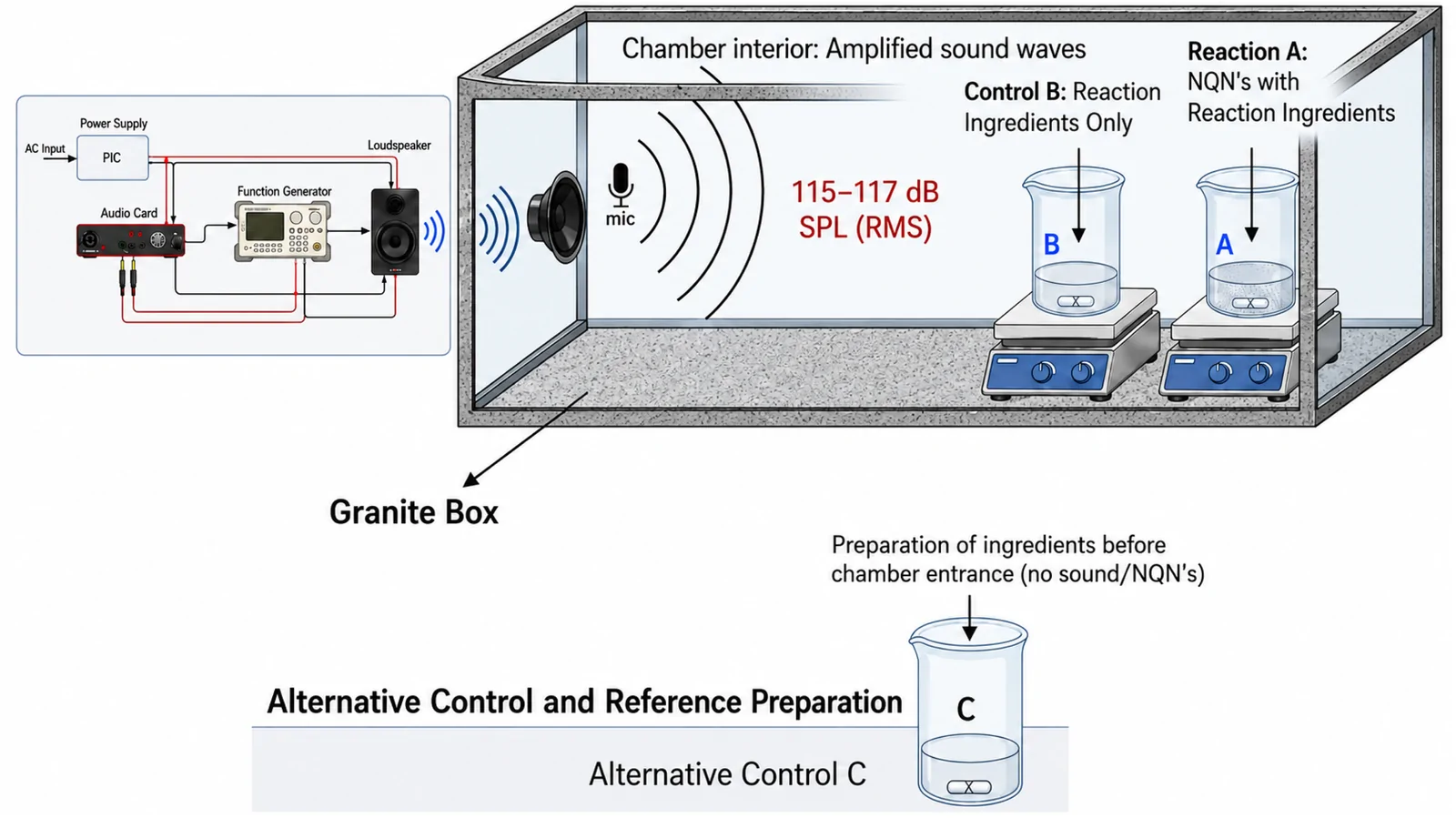
Diagram of sound waves amplification technique as a source of stress on natural quartz nanoparticle at ambient temperature.

The data show that the combination of amplified acoustic irradiation and NQNPs is markedly more efficient than acoustic irradiation alone: without NQNPs the reactions required four- to twelve-fold longer times and gave lower yields (Tables 1 and 2). Under conventional stirring at room temperature, without NQNPs and without acoustic irradiation, no product was detected even after standing overnight. A control containing NQNPs but no acoustic irradiation was not run separately, so the contribution of NQNPs in the absence of the acoustic field was not evaluated.

**Table 2:** Comparative study of reaction time and yield for the synthesis of pyridine derivatives (**4**, **5**, **6**) under amplified acoustic activation, conventional stirring, and the reported method

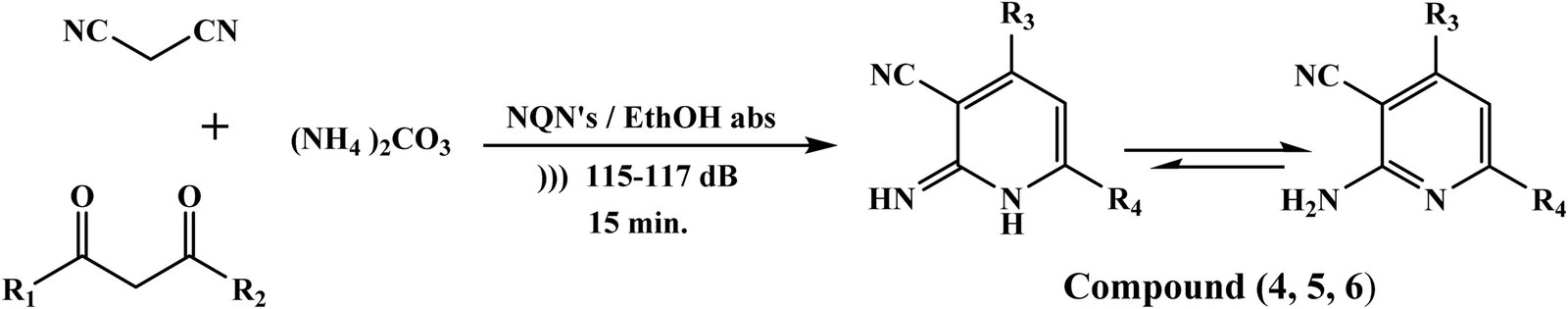																			
Entry	*R*_1_	*R*_2_	Product	Time (min)				Yield%				Catalyst				Reaction conditions			
				This study method			Ref. no.	This study method			Ref. no.	This study method			Ref. no.	This study method			Ref. no.
				*A*	*B*	*C* over night	38 and 39	*A*	*B*	*C*	38 and 39	*A*	*B*	*C*	38 and 39	*A*	*B*	*C*	38 and 39
1	CH_3_	CH_3_	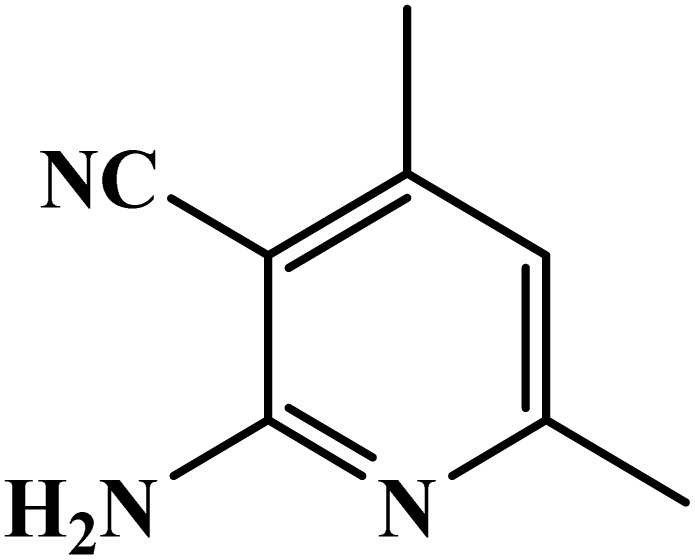	15	60	NR	60 (ref. 38)	92	60	0	73 (ref. 38)	NQN's	—	—	—	Amplified sound waves/NQN's	Amplified sound waves	Standing only at RT	Reflux in water bath^38^
2	OH	H	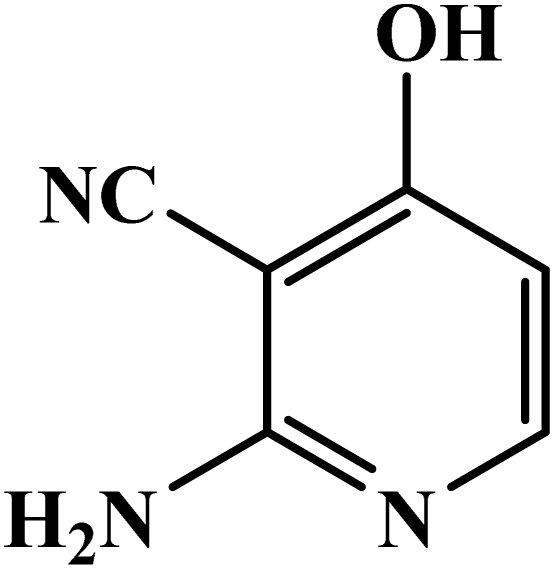	15	60	NR	120	95	64	0	83	NQN's	—	—	—	Amplified sound waves/NQN's	Amplified sound waves	Standing only at RT	Ammonia/water/2 h/Heating/reflux
3	CH_3_	H	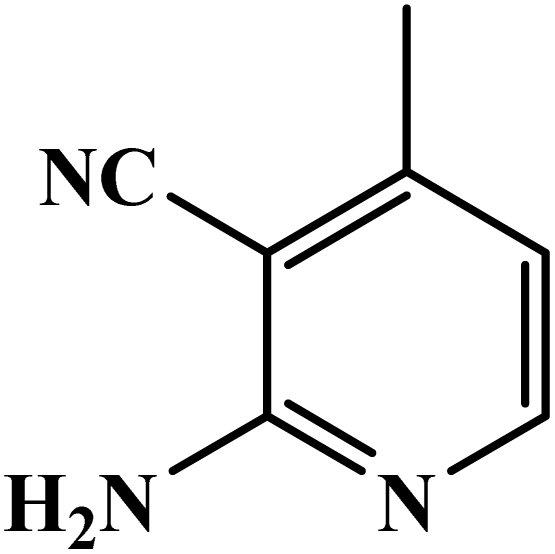	15	180	NR	180 (ref. 39)	94	66	0	50 (ref. 39)	NQN's	—	—	—	Amplified sound waves/NQN's	Amplified sound waves	Standing only at RT	Heated for 3 h at 150 °C (ref. 39)

Compared with the literature protocol reported by Tamaddon and Maddah-Roodan,^15^ which required heating at 80 °C for approximately 240–300 min, the present strategy reduced the reaction time by more than 90% while maintaining comparable or slightly higher isolated yields under completely ambient conditions.

The FT-IR spectrum displayed a broad hydroxyl absorption at 3290–2798 cm^−1^, a characteristic nitrile stretching band at 2215 cm^−1^, and a C=N absorption at 1624 cm^−1^, confirming formation of the pyridine nucleus for compound 1. The ^1^H NMR spectrum exhibited two singlets at *δ* 2.22 and 2.30 ppm corresponding to the two methyl groups located at C-4 and C-6. A singlet at *δ* 6.15 ppm was assigned to the isolated pyridine proton, while the hydroxyl proton appeared as a highly deshielded singlet at *δ* 12.10 ppm, disappearing upon D_2_O exchange, indicating strong intramolecular hydrogen bonding. The ^13^C NMR spectrum showed eight carbon signals in agreement with the proposed structure, including methyl carbons around 19–21 ppm, the nitrile carbon at 116.54 ppm, and oxygenated aromatic carbons between 152–162 ppm.

Replacement of acetylacetone with ethyl acetoacetate produced compound 2, whose FT-IR spectrum retained the characteristic OH, C≡N, and C=N absorptions. The ^1^H NMR spectrum exhibited a methyl singlet at *δ* 1.98 ppm, a pyridine proton at *δ* 5.02 ppm, and a broad resonance integrating for two hydroxyl groups at *δ* 10.05 ppm, confirming formation of the dihydroxylated pyridine derivative. The ^13^C NMR spectrum displayed signals corresponding to one methyl carbon, the nitrile carbon at 116.72 ppm, and three oxygen-bearing aromatic carbons between 155 and 166 ppm, fully supporting the assigned structure.

The trihydroxylated derivative 3, obtained from diethyl malonate, exhibited a broad OH stretching band in the FT-IR spectrum together with the nitrile absorption at 2264 cm^−1^ and the pyridine C=N band at 1589 cm^−1^. Its ^1^H NMR spectrum contained a singlet at *δ* 6.13 ppm assigned to the pyridine proton and three exchangeable hydroxyl resonances appearing at *δ* 10.94, 12.18, and 13.48 ppm, indicating three chemically distinct hydroxyl groups. The ^13^C NMR spectrum showed characteristic oxygenated aromatic carbons between 165–171 ppm, confirming complete aromatization of the pyridine ring. Overall, the spectroscopic data agree well with the proposed molecular structures and are fully consistent with previously reported hydroxynicotinonitrile derivatives.

A plausible mechanism for the synthesis of compounds 1–3 is illustrated in Fig. 6. Initially, ammonium carbonate, whose decomposition is promoted by the acoustic field, releases ammonia and carbonate species. Ammonia subsequently reacts with ethyl cyanoacetate through aminolysis to generate cyanoacetamide, which has previously been identified as the key intermediate in the Guareschi–Thorpe reaction. Simultaneously, carbonate ions maintain a mildly basic buffered medium that facilitates deprotonation of the active methylene group. The activated cyanoacetamide then undergoes Knoevenagel-type condensation with the corresponding 1,3-dicarbonyl compound to generate an α,β-unsaturated intermediate. The carbonyl groups are further activated through hydrogen-bond interactions with surface silanol (Si–OH) groups present on natural quartz nanoparticles, thereby increasing their electrophilicity and facilitating nucleophilic attack. Subsequently, intramolecular cyclization occurs through nucleophilic attack of the amino nitrogen onto the activated carbonyl center, producing a cyclic dihydropyridine intermediate. Proton transfer and dehydration then generate the aromatic hydroxypyridine framework. Finally, keto–enol tautomerization affords the thermodynamically stable hydroxynicotinonitrile derivatives (1–3).

**Fig. 6:**
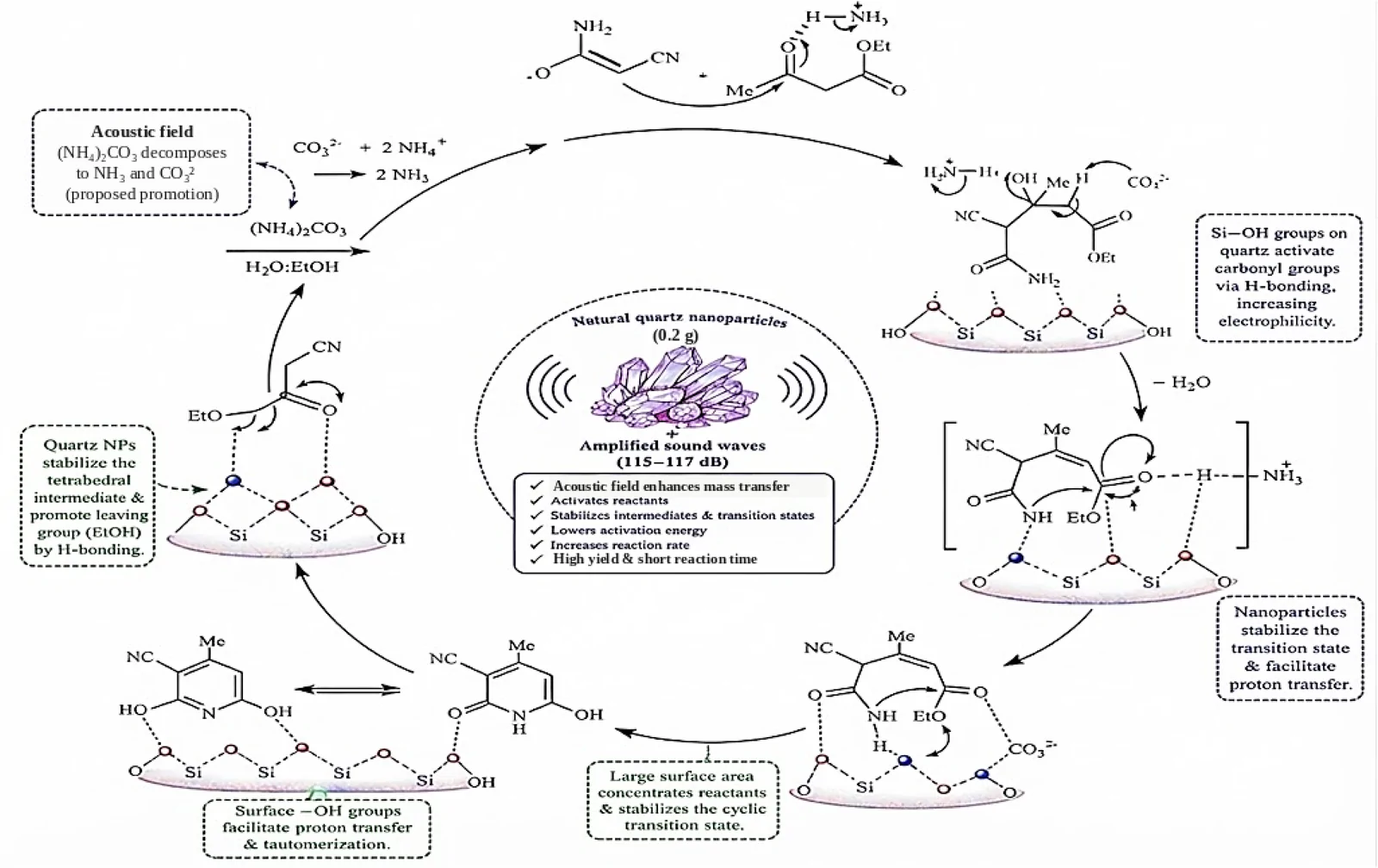
Plausible reaction mechanism illustrating the catalytic role of natural quartz nanoparticles under amplified sound waves.

Natural quartz nanoparticles are proposed to perform several catalytic functions throughout the reaction sequence. Their surface is rich in silanol (Si–OH) groups capable of forming reversible hydrogen bonds with carbonyl oxygen atoms. These interactions increase carbonyl electrophilicity and lower the activation energy of nucleophilic addition. The large specific surface area provides abundant adsorption sites that concentrate reactants at the catalyst surface, thereby increasing effective molecular collisions. Moreover, hydrogen bonding stabilizes high-energy tetrahedral intermediates and cyclic transition states while facilitating proton-transfer events required during cyclization and tautomerization. Unlike conventional homogeneous catalysts, NQNPs remain chemically stable, inexpensive, and readily recoverable, making them highly attractive heterogeneous catalysts.

The role of the amplified acoustic field (115–117 dB, 400–500 Hz) is more difficult to define. At these audible frequencies and sound pressures (rms ≈ 14 Pa), classical inertial cavitation, which typically requires ultrasonic frequencies and acoustic pressure amplitudes of the order of 10^5^ Pa, is not expected and was not verified in this work; the acceleration is therefore not attributed to cavitation hot spots. In our experiments the nanoparticles compacted into a hard layer at the bottom of the vessel and no suspended particles were observed. The acoustic field is thus proposed to act (i) by promoting mixing and mass transfer at the solid–liquid interface, (ii) by facilitating the decomposition of ammonium carbonate to ammonia, which supplies the nitrogen source, and (iii) by exerting a cyclic mechanical stress on the compacted quartz layer, which may polarize the non-centrosymmetric quartz lattice through the direct piezoelectric effect (see the Piezocatalytic section). The relative contributions of these effects have not been separated experimentally.

The piezoelectric contribution should therefore be regarded as a working hypothesis. It is supported by the measured *d*_33_ of the compacted powder, by the much shorter reaction times obtained with quartz than with amorphous silica or sand under the same acoustic conditions (qualitative preliminary observations), and by the marked acceleration of the acoustically driven reaction in the presence of NQNPs. It is not supported by direct spectroscopic evidence: ESR spin-trapping experiments (DMPO/TEMP), *in situ* IR/NMR monitoring and piezoresponse force microscopy were not available. Consequently, it is not established whether the activation proceeds by direct charge transfer that polarizes reactant bonds or indirectly through surface-charge-induced formation of reactive species such as ˙OH or ˙O_2_^−^, and the physicochemical (silanol-based) interactions described above remain an important contributing factor. Radical-scavenger experiments and the other controls listed under “Scope, limitations and outlook” would be the next steps to address this point.

To further demonstrate the versatility of the developed protocol, ethyl cyanoacetate was replaced with malononitrile while maintaining the same reaction conditions (Methods A–C). Under these conditions, the corresponding pyridine derivatives 2-amino-4,6-dimethylnicotinonitrile (4), 2-amino-4-hydroxynicotinonitrile (5), and 2-amino-4-methylnicotinonitrile (6) were successfully synthesized in good to excellent yields, Table 2. The reactions proceeded efficiently through a three-component condensation of malononitrile, ammonium carbonate, and the appropriate 1,3-dicarbonyl compound under amplified acoustic irradiation in the presence of natural quartz nanoparticles (NQNPs).

A comparison between the different synthetic methods clearly demonstrates the advantage of the NQNP-assisted acoustic protocol. Method A (amplified acoustic irradiation in the presence of NQNPs) afforded the desired products within only 15 min, whereas Method B (amplified acoustic irradiation without catalyst) required 60–180 min, depending on the substrate. In contrast, Method C (conventional stirring at room temperature) failed to produce the target compounds even after prolonged reaction times, indicating that simple mechanical mixing is insufficient to overcome the activation barrier of the multicomponent condensation.

The isolated yields of compounds 4–6 under Method A were similar (4 : 92%; 5 : 95%; 6 : 94%). These small differences are within the range expected from experimental variation and are not interpreted mechanistically. When compared with previously reported procedures, the present methodology shows clear advantages in reaction time. Earlier methods required 60 min under ultrasonic irradiation without catalyst, 6 h of stirring at room temperature, or reflux/heating at elevated temperatures for several hours, often providing only moderate to good yields.^38,39^ In contrast, the present protocol combines natural quartz nanoparticles with acoustic activation, reducing the reaction time to only 15 min with yields of 92–95%. These findings indicate a beneficial combined effect of heterogeneous catalysis and acoustic activation in promoting the rapid synthesis of nicotinonitrile derivatives.

The structures of compounds 4–6 were confirmed by melting point determination and spectroscopic analyses (IR, ^1^H NMR, and ^13^C NMR). In the IR spectra, characteristic absorption bands corresponding to the amino group appeared at 3491–3330 cm^−1^, while the nitrile functionality showed a strong band around 2218 cm^−1^, confirming the incorporation of the cyano group. Compound 5 additionally exhibited a broad hydroxyl stretching vibration at 3444 cm^−1^, consistent with the presence of the 4-hydroxypyridine tautomer.

The ^1^H NMR spectra further supported the proposed structures. Compound 4 displayed two singlets at *δ* 2.22 and 2.30 ppm corresponding to the two methyl substituents, together with a singlet at *δ* 6.15 ppm assigned to the pyridine proton and a broad exchangeable NH_2_ signal at *δ* 6.70 ppm. Compound 5 exhibited two aromatic pyridine protons at *δ* 7.48–7.54 ppm, an amino signal at *δ* 6.46 ppm, and a strongly deshielded hydroxyl proton at *δ* 10.67 ppm, indicative of intramolecular hydrogen bonding and keto–enol tautomerism. Similarly, compound 6 showed a methyl singlet at *δ* 2.20 ppm, aromatic pyridine protons at *δ* 7.80–7.86 ppm, and an exchangeable NH_2_ resonance at *δ* 6.96 ppm.

The ^13^C NMR spectra were also fully consistent with the assigned structures. The nitrile carbon resonated at approximately *δ* 116 ppm, while the pyridine ring carbons appeared within the expected range of *δ* 108–175 ppm depending on the nature of the substituent. Compound 4 displayed two methyl carbon signals at *δ* 19.34 and 21.07 ppm, whereas compound 6 exhibited a single methyl carbon resonance at *δ* 24.62 ppm. The downfield carbon signals observed for compound 5 reflected the electron-withdrawing influence of the hydroxyl-substituted pyridine framework.

Compound 7 was successfully synthesized *via* a one-pot multicomponent Guareschi–Thorpe condensation using equimolar amounts of ethyl cyanoacetate and malononitrile in the presence of ammonium carbonate under the optimized reaction conditions. Replacing one equivalent of ethyl cyanoacetate with malononitrile significantly altered the reaction pathway, leading to the formation of a highly functionalized pyridine derivative bearing two amino groups, one hydroxyl group, and a nitrile functionality, Scheme 1.

**Scheme 1:**
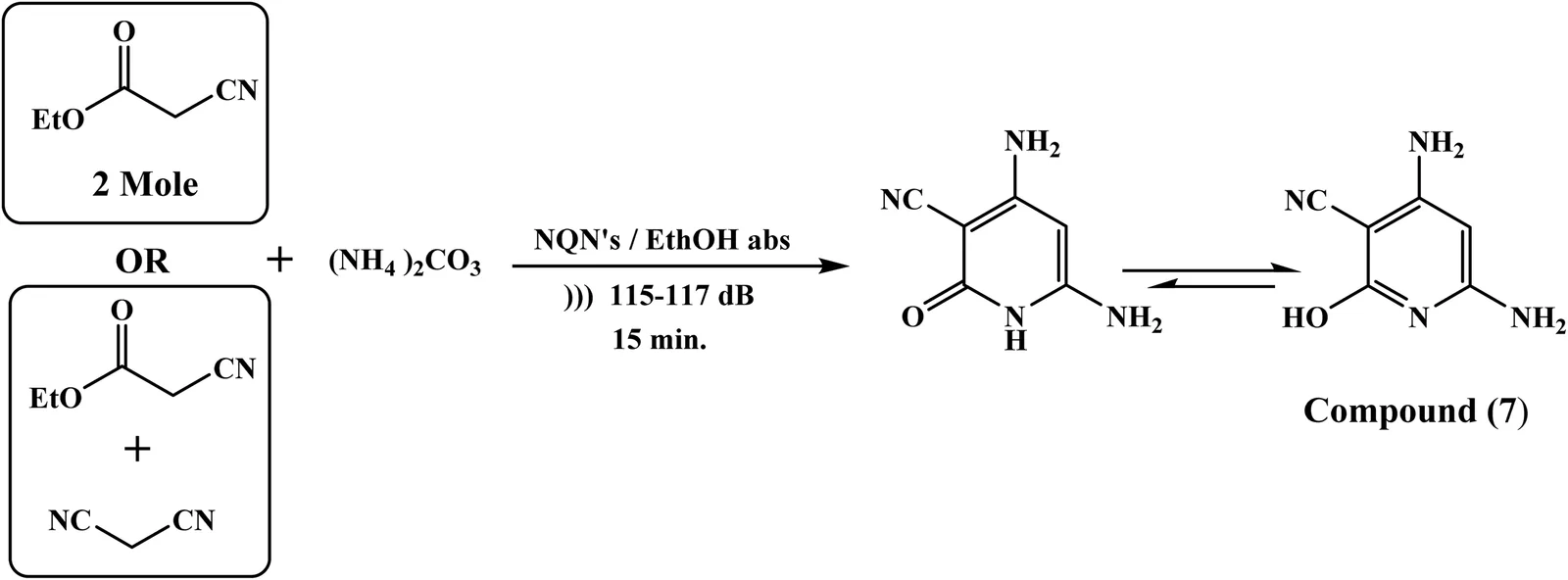
One-pot multicomponent Guareschi–Thorpe condensation leading to compound 7.

The reaction is proposed to proceed through the initial formation of β-enaminonitrile intermediates generated by the reaction of the active methylene compounds with ammonia released from ammonium carbonate. Subsequent intermolecular condensation, followed by intramolecular cyclization and aromatization, afforded the substituted nicotinonitrile derivative. The strong electron-withdrawing nature of the nitrile groups facilitates cyclization and promotes the formation of the pyridine nucleus.

Compound 7 was isolated as yellow crystals with a melting point of 180–182 °C, indicating good purity. Its structure was confirmed by spectroscopic analyses. The IR spectrum exhibited a broad absorption band at 3421 cm^−1^ corresponding to the hydroxyl group, while bands at 3348 and 3310 cm^−1^ were assigned to the asymmetric and symmetric stretching vibrations of the amino groups. The characteristic nitrile stretching vibration appeared at 2189 cm^−1^. The ^1^H NMR spectrum displayed two singlets at *δ* 4.34 and 7.31 ppm, corresponding to two chemically non-equivalent amino groups, reflecting their different electronic environments within the pyridine ring. A singlet at *δ* 7.85 ppm was assigned to the only aromatic proton of the pyridine ring, whereas the downfield broad singlet at *δ* 12.24 ppm confirmed the presence of a hydrogen-bonded hydroxyl proton. The ^13^C NMR spectrum showed six distinct carbon resonances consistent with the expected substituted pyridine skeleton, including signals attributable to the nitrile carbon and oxygen- and amino-substituted sp^2^ carbons.

### Piezocatalytic and electroacoustic behavior of natural quartz nanoparticles

Natural quartz is a non-ferroelectric piezoelectric material that exhibits a stable piezoelectric response when subjected to mechanical stress. Although its piezoelectric properties *d*_33_ = 3.3 ± 0.2 pC/N using (Quasi-Static *d*_33_ Meter) are generally less tunable than those of ferroelectric materials, quartz possesses several advantages Fig. 7, including excellent chemical stability, environmental compatibility, low cost, and natural abundance, making it an attractive material for catalytic applications.^40–44^

**Fig. 7:**
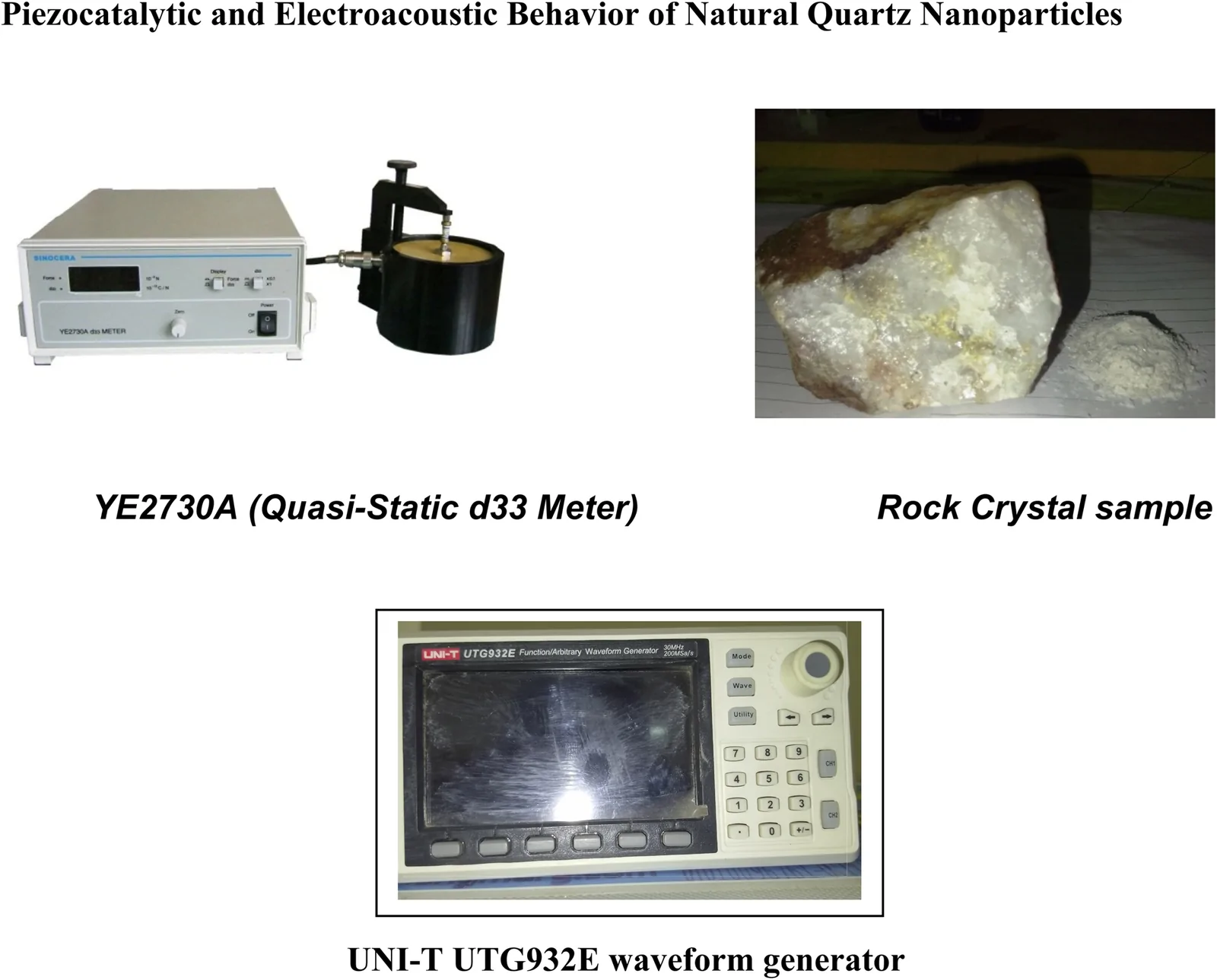
Natural rock crystal sample and experimental setup used for piezoelectric characterization, including the YE2730A quasi-static *d*_33_ meter and UNI-T UTG932E waveform generator.

In the present study, natural quartz nanoparticles (NQNPs) demonstrated remarkable catalytic activity^45,46^ in the one-pot synthesis of the target heterocyclic compounds. The incorporation of NQNPs under amplified sound waves significantly accelerated the reactions, reducing the reaction time four- to twelve-fold compared with the corresponding reactions performed in the absence of the catalyst while simultaneously improving the product yields.

The enhanced catalytic performance is proposed to arise from the synergistic combination of the piezoelectric nature of quartz and the applied acoustic field. When mechanical stress generated by amplified sound waves is transferred to the quartz nanoparticles, deformation of the crystal lattice may induce surface polarization and transient piezoelectric charges. These surface charges can facilitate charge separation, promote reactant adsorption and activation, and stabilize charged intermediates and transition states during the reaction pathway, thereby lowering the activation energy and accelerating product formation.

To generate the required mechanical excitation, acoustic waves were produced using a function generator operating in the frequency range of 400–500 Hz. The acoustic energy was confined within a custom-designed granite resonance chamber (13 × 14 × 9 cm), resulting in amplified sound intensities of approximately 115–117 dB. Under these conditions the nanoparticles compacted into a hard layer firmly attached to the bottom of the vessel (an iron spatula was needed to detach it) and no suspended particles were observed; the mechanical stress is therefore applied to a compacted layer rather than to a dispersed suspension. This compaction was not observed with amorphous chromatographic silica or commercial sand in the preliminary trials.

This electroacoustic-assisted catalytic strategy enabled the synthesis of the desired products under ambient conditions without external heating and with a lower estimated energy input than the thermal benchmark (see “Energy input and reaction mass metrics”). Nevertheless, the quantitative relationship among acoustic frequency, sound intensity, piezoelectric charge generation, and catalytic performance has not yet been established. Further experimental and theoretical investigations are therefore required to elucidate the underlying piezocatalytic mechanism and optimize the electroacoustic response of natural quartz nanoparticles for organic synthesis. We used a natural rock in this research a quartz type of rock crystal sample. Which is crushed manually at first then we used ball mill for fine grinding process.
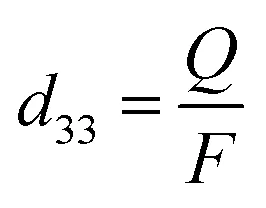
where *Q* is the charge generated by the sample.

The calculation of *d*_33_ value for SiO_2_ quartz (rock crystal) sample

• Sample diameter = 1.5 cm

• Thickness = 2.1 mm

• Applied force = 26.2 × 10^−3^ N

• Measured *d*_33_ values:

• 3.2 × 10^−12^ C/N

• 3.4 × 10^−12^ C/N

Then the values are: *d*_33_ = 3.2 × 10^−12^ C/N = 3.2 pC/N

And *d*_33_ = 3.4 × 10^−12^ C/N = 3.4 pC/N

Average *d*_33_
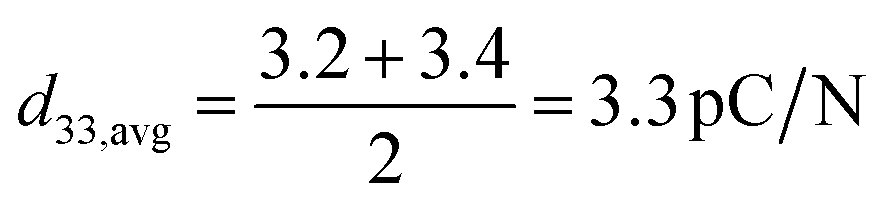


So *d*_33_ = 3.3 ± 0.2 pC/Nwhere ±0.2 pC/N represents the half-range for two measurements.

The value of 3.3 ± 0.2 pC/N is an effective *d*_33_ measured with a quasi-static *d*_33_ meter on pressed and sintered discs prepared from the natural quartz powder (1.5 cm diameter, about 2 mm thick; two measurements); it is not a single-crystal coefficient. For single-crystal α-quartz (point group 32) the *d*_33_ component is zero by symmetry and the principal coefficient is *d*_11_ ≈ 2.3 pC/N, so the measured value should be regarded as a phenomenological, orientation-averaged response of the compacted polycrystalline powder. It is about two orders of magnitude lower than the values of ferroelectric ceramics such as BaTiO_3_ and PZT. Piezoresponse force microscopy (PFM) was not available, and the value rests on only two measurements.

An order-of-magnitude estimate of the piezoelectric potential was made for the compacted catalyst layer (0.2 g; area ≈1.77 × 10^−4^ m^2^; assumed thickness ≈1.5 mm; *ε*_r_ ≈ 3.8). With an rms sound pressure of 14.16 Pa (peak ≈20 Pa; 117 dB), the force on the layer is *F* = *P*·*A* ≈ 2.5–3.5 mN, the generated charge is *Q* = *d*_33_·*F* ≈ 0.8–1.2 × 10^−14^ C, the layer capacitance is *C* = *ε*_0_*ε*_r_*A*/*t* ≈ 4.0 × 10^−12^ F, and the open-circuit potential is *V* = *Q*/*C* ≈ 2–3 mV. This macroscopic value is small. Local stress concentration at inter-particle contacts in the compacted layer could produce higher local fields, but this was neither modelled nor measured, and the estimate should not be read as the potential at the particle–solution interface. For comparison, the same approach applied to the 2 mm sintered disc under the 26.2 mN test force gives ≈25 mV.

### Acoustic chamber, sound pressure and frequency selection

Granite box dimentions are 9 × 14 × 13 cm with wall thickness 1.5 cm and a tiny hole of diameter 10 mm (at the face of 13 × 9) as an exit hole for the speaker's wire.

1. (Resonance & wave accumulation): granite has an incredibly high acoustic impedance that mean over 99.9% of sound waves are reflected back to the box cavity rather than box walls penetration.

2. (Acoustic suspension/compression) of the speaker that the speaker act as air compressor and vacuum at the same time which convert the kinetic energy of the speaker membrane to continuous pressure (acoustic compliance) which in turn increase the vibration significantly in comparison of the open air situation.

3. The soft and polished internal box walls prevent scattering and minimizes viscous boundary layer thermal losses, keeping the sound energy trapped. The selection of granite resonance box enabled us to acheave remarkable sound amplification process as the following:

#### Choice of material

In preliminary trials, boxes made of acrylic and of wood vibrated under the applied sound intensity, moved, and spilled their contents. The granite chamber remained stable and, owing to its high acoustic impedance, kept the sound energy inside the cavity.

#### Sound pressure and stress

The sound pressure level inside the chamber was measured with a sound-level meter (CROWN CT 44097) as 115–117 dB. With *p*_0_ = 2 × 10^−5^ Pa, *p*_rms_ = *p*_0_ × 10^(*L*_p_/20)^, which gives *p*_rms_ ≈ 11.2 Pa (115 dB) and ≈14.2 Pa (117 dB); the corresponding peak pressure of a sinusoidal wave is √2 × *p*_rms_ ≈ 16–20 Pa. The nominal acoustic stress on the vessel is therefore about 1–2 × 10^−5^ MPa. The local stress transmitted to individual contact points within the compacted quartz layer cannot be derived from these data and was not measured.

#### Amplification

The sound level of the source outside the chamber was not recorded, so the gain provided by the chamber is described qualitatively (the sealed enclosure lets the speaker cone compress the trapped air, *i.e.* acoustic compliance) and is not quantified. No acoustic finite-element simulation or spatial mapping of the sound pressure inside the vessel was carried out; the pressure-distribution sketch in Fig. 5 is a qualitative illustration only.

#### Frequency selection

The 400–500 Hz range was selected empirically within the audible range and was not derived from a standing-wave or modal analysis of the chamber or of the nanoparticles. For orientation, the wavelength at 450 Hz (≈0.76 m) is much larger than the chamber dimensions, so the enclosure behaves largely as a sealed pressure cavity, and a simple rigid-cavity estimate (*f* = *c*/2 *L*, *L* = 14 cm) places the first axial standing-wave mode near 1.2 kHz, above the frequency range used. A quantitative model of the standing-wave field is under development.

### X-ray fluorescence

XRF spectrometry is particularly valuable because of its ability to rapidly provide a high-resolution assessment of relative variations of most Earth elemental compositions.^47^ XRF spectrometry is based on the wavelength-dispersive principle, which states that individual atoms emit a relative abundance of X-ray photons of energy or wavelength feature that can be estimated.^48^ This technique has been applied in the examination of Earth materials from a range of settings and environments.^49^ The presence of aluminum ions Al^3+^ in quartz minerals also causes a strong increase in the quality factor of electro-elastic vibrations of piezoelectric particles, which may also may be associated with a change in the surface charge of quartz particles,^47^Table 3.^50^

**Table 3:** X-ray fluorescence

Compound formula	Concentration (wt%)
Na_2_O	0.017
MgO	0.021
Al_2_O_3_	0.287
SiO_2_	98.7
P_2_O_5_	0.014
SO_3_	0.024
K_2_O	0.107
CaO	0.275
Fe_2_O_3_^III^	0.071
ZnO	0.016
F	0.034
Cl	0.034
L.O.I	0.4

The XRF analysis shows that the natural quartz is 98.7 wt% SiO_2_ with minor Al_2_O_3_ (0.287%), CaO (0.275%), K_2_O (0.107%) and Fe_2_O_3_ (0.071%). At these low levels a substantial catalytic role of Fe or Al sites is considered unlikely, but a synergistic effect of trace impurities cannot be excluded; for example, Al^3+^ has been reported to increase the quality factor of the electro-elastic vibrations of quartz particles.^47^ The activity of NQNPs was not compared with that of synthetic, phase-pure quartz nanoparticles or with other piezoelectric minerals such as berlinite (a rare mineral that was not available); the aim of this work was to use an abundant, inexpensive and easily handled natural mineral.

#### Preliminary control observations

In preliminary trials, chromatographic-grade (amorphous) silica and commercial sand were tested under the same acoustic conditions. With NQNPs the reaction mixture was clear and turned faintly yellow, and TLC showed conversion after 10–25 min, whereas with chromatographic silica or sand the mixture remained turbid and only traces of product were detected after 240 min and after standing overnight. Both amorphous silica and natural quartz carry surface silanol (Si–OH) groups, so hydrogen-bond activation of the reactants should operate in both cases; the much shorter reaction time with quartz therefore points to an additional factor, which we tentatively associate with the piezoelectric response, although this is not proven. These observations are qualitative: the particle size and surface area of the silica and sand were not matched to those of the NQNPs, quantitative yields were not determined, and controls with NQNPs without acoustic irradiation or with NQNPs at sub-threshold sound levels (<100 dB) were not carried out. The effect of catalyst loading was also examined: increasing the amount of NQNPs to 1.5, 2 and 2.5 times the standard amount (0.20 g) changed the reaction time by only ±0.5–1.5 min, indicating that under these conditions the reaction time is not limited by the catalyst loading.

### Catalyst recyclability

The natural quartz nanoparticles were separated after the reaction finished by filtering, washing with absolute ethanol and then dried over vacuum. The catalyst was effectively recycled over six successive cycles with no significant weight loss. The reusability of the NQN's catalyst was further evaluated over six consecutive reaction cycles under the optimized conditions. As shown in Table 4, the catalyst maintained nearly consistent catalytic activity, with only a slight decrease in yield from 98% in the first cycle to 95% in the sixth cycle. This minimal reduction confirms the catalyst's stability and recyclability under the reaction conditions. The use of NQN's is due to Quartz is a hard mineral and it also impacts good corrosion resistance and high chemical stability.^44^ NQN's has facile recovery and reuse which demonstrate their advantages in synthesis chemistry.

**Table 4:** Catalyst recycling

Cycle	Yield (%)
1	98
2	98
3	97
4	≈97
5	96
6	95

### Effect of solvent on reaction

To set the optimal reaction conditions, solvent optimization was carried out under the same reaction conditions. Among the tested solvents, ethanol afforded the highest yield (≈98%), methanol gave a lower yield (88%), and water showed lower yield product formation. The solvent-free condition (neat) was also less effective (18%). These results indicate that ethanol is the most suitable solvent for such reactions Table 5.

**Table 5:** Effect of solvent

Entry	Solvent	Yield (%)
1	Ethanol (*ε* ≈ 24.5)	≈98
2	Methanol (*ε* ≈ 32.7)	88
3	Water (*ε* ≈ 80)	75
4	Neat (no solvent)	18

The yield does not follow the dielectric constant (*ε* at 25 °C: ethanol ≈24.5, methanol ≈32.7, water ≈80) or the polarity of the solvent in a simple monotonic way (98%, 88% and 75%, respectively), and the low yield under neat conditions (18%) shows that a liquid medium is needed for mixing and mass transfer. Reactant and product solubility (the product precipitates from ethanol), viscosity and the ability to solvate ions may therefore be as important as polarity. How the solvent dielectric properties influence the piezoelectric field at the particle surface, and whether the solvent itself is activated, was not investigated; solvents of lower polarity such as acetonitrile or toluene were not tested.

### Energy input and reaction mass metrics

#### Energy input

The electrical energy of the acoustic set-up was estimated from the nominal ratings of the speaker (5 W) and of the function generator (5 V/2 A supply, 10 W); no separate amplifier was used and the power actually drawn was not measured, so 15 W is an upper-bound estimate. For a 1 mmol reaction *E* = *P*·*t*. The thermal benchmark^15^ (80 °C, 240–300 min) was estimated assuming a hot plate consuming an average of 20 W (range 15–30 W) without accounting for heat losses, which is a conservative (low) estimate for a laboratory hot plate. On this basis the electroacoustic protocol requires about 13.5–22.5 kJ mmol^−1^, compared with about 288–360 kJ mmol^−1^ for the thermal protocol, *i.e.* roughly 21–27 times less for compounds 1 and 2 (Table 5). Acoustic irradiation alone (Method B) is estimated at 54 kJ mmol^−1^, about 5–7 times less than the thermal benchmark, Table 6.

**Table 6:** Estimated energy input per mmol of substrate (1 mmol scale; multiply by 1000 for kJ mol^−1^)

Protocol	Conditions	Time (min)	Power (W)	Energy (kJ mmol^−1^)
Method A, compounds 1 and 2	Sound + NQNPs, rt	15	15 (nominal)	13.5
Method A, compound 3	Sound + NQNPs, rt	25	15 (nominal)	22.5
Method B, compounds 1 and 2	Sound only, rt	60	15 (nominal)	54
Method B, compound 3	Sound only, rt	180	15 (nominal)	162
Thermal,^15^ compound 1	80 °C, hot plate	300	20 (15–30, assumed)	360 (270–540)
Thermal,^15^ compound 2	80 °C, hot plate	240	20 (15–30, assumed)	288 (216–432)

#### Reaction mass metrics

The process mass intensity (PMI = total mass of inputs/mass of product) and the *E*-factor ((total input − product)/product) were calculated for the reaction step of compound 1 on the 1 mmol scale (98% yield, 145 mg), counting all reagents and the reaction solvent. Work-up solvents (ethanol used for washing and recrystallization) were not quantified and are excluded for both protocols; the catalyst (0.20 g) was recovered and reused over six cycles and is excluded (counting it once would raise the PMI from 56.5 to 57.8). The benchmark^15^ uses 1 mmol of each reactant, 2 mmol of ammonium carbonate and 2 mL of EtOH/H_2_O (1 : 1) at 80 °C (95% yield, 141 mg). The reaction-step PMI and *E*-factor of the present protocol are therefore about 3.6 times higher than those of the benchmark (Table 6), mainly because 10 mL of ethanol (96% of the input mass) was used. The solvent volume was not optimized, and reducing it is a target for future work. The present method thus offers a lower estimated energy input and a shorter reaction time, but not a lower material intensity, Table 7.

**Table 7:** Reaction-step mass metrics for compound 1 (1 mmol scale, work-up excluded)

Metric	This work (Method A)	Thermal benchmark^15^
Total input mass, excluding catalyst (g)	8.20	2.19
Product mass (mg)	145	141
PMI	56.5	15.6
*E*-Factor	55.5	14.6
Solvent share of input mass	96%	82%

### Scope, limitations and outlook

(i) The scope of the study is limited to two active methylene nitriles (ethyl cyanoacetate, malononitrile) and three simple, mostly liquid 1,3-dicarbonyl partners chosen to simplify mixing, plus the mixed product 7. Substituted aryl-1,3-dicarbonyl compounds, substrates bearing groups sensitive to radicals or strong local electric fields (*e.g.* reducible halides, olefins) and other multicomponent reactions were not examined. (ii) The nanoparticles are difficult to remove completely and contaminate the crude product, particularly in the NMR spectra; repeated filtration and recrystallization were needed. (iii) The speaker overheated during continuous operation and had to be paused, and electrical connections were a source of interruptions; although no external heating was applied, a contribution from mild warming cannot be strictly excluded, and a temperature-logged control is recommended. (iv) The reactions were run on a 1 mmol scale in 10 mL of solvent in a 9 × 13 × 14 cm chamber. Scaling to volumes above 100 mL would require a larger chamber, a more powerful transducer, and a demonstration that the sound pressure and the stress on the catalyst layer remain uniform; none of this has been done. The main practical challenges identified were obtaining suitable granite and electrical components that do not overheat. (v) No kinetic analysis was performed; because the Guareschi–Thorpe reaction proceeds through several consecutive steps (Knoevenagel condensation, imine/enamine formation, Michael addition, cyclization, dehydration/aromatization), no single rate-determining step could be identified and rate constants were not determined. (vi) Direct evidence for the mechanism (ESR spin trapping, *in situ* IR/NMR monitoring, PFM, DFT calculations) was not available. (vii) Nitrogen sources other than ammonium carbonate (NH_4_Cl, NH_4_OAc, ammonia) were not explored. Future work should address these points, in particular controls with NQNPs alone and at low sound pressure, purified or synthetic quartz, radical scavengers, and temperature-logged experiments.

## Experimental

### Chemicals and reagents

All chemicals and reagents were procured from Aldrich Chemical Co., Inc. (WI, USA) and used without further purification. Thin-layer chromatography (TLC) was utilized to monitor the synthesized compounds, employing precoated silica gel sheets (Merck 60 F254). Spots were visualized under UV light at 254 nm. Melting points were determined using an Electrothermal IA 9100 apparatus and are uncorrected. IR spectra (KBr) were measured on a Nexus 670 FTIR Nicolet, Fourier transform infrared spectrometer. The ^1^H and ^13^C-NMR spectroscopy were determined with a JEOLJNM-LA 400 MHz spectrometer. The chemical shifts are expressed on the *δ* (ppm) scale using tetramethylsilane as the standard reference. Elemental analysis determined on a PerkinElmer 240 (microanalysis), Microanalysis Center, Cairo University, Cairo, Egypt. (E. Merck). The sound intensity was detected buy (CROWN – CT 44097). UNI-T UTG932E is a compact, dual-channel function and arbitrary waveform generator that runs on a 5 V DC/2 A power supply input. The XRD for NQN's was measured by desert research center in Cairo. The FESEM images and the EDX spectrum of the NQNs were recorded on a Quanta FEG 250 (Thermo Fisher Scientific) at the National Research Centre, Egypt. Sinocera Piezotronics, Inc. for piezoelectric coefficient measuring and XRF analysis in Institution of Metallurgy Research in Hellwan–Cairo.

#### Safety precautions

Quartz nanoparticles are respirable crystalline silica and must be handled as a hazardous powder: weigh and transfer them in a fume hood or ventilated enclosure; wear a certified respirator (N95/FFP3), gloves and eye protection; avoid dry sweeping (use wet wiping or a HEPA vacuum for spills); and dispose of the recovered and waste catalyst as hazardous solid waste. Because sound levels of 115–117 dB were generated inside the chamber, the sound level outside the chamber should be checked and hearing protection used if necessary, and the speaker and electrical connections should be monitored for overheating. Detailed guidelines are given in the SI.

### Experimental procedures

#### General procedure for compounds (1, 2, 3)

##### Method A: amplified acoustic activation in the presence of natural quartz nanoparticles

Ethyl cyanoacetate (1 mmol), ammonium carbonate (1 mmol), and the appropriate 1,3-dicarbonyl compound (1 mmol), namely acetylacetone, ethyl acetoacetate, or diethyl malonate, were introduced into a reaction vessel containing 10 mL absolute ethanol and natural quartz nanoparticles (NQNPs, 0.20 g). The container was set at the corner of granite box. The box contained also a 5 watt – 4 Ω speaker. The speaker is connected to function generator UTG 932E of 2 amp, and 5 Volt. The speaker gave sound waves (400–500 Hz) for 15–25 min till the reaction end. The reaction mixture was exposed to amplified sound waves (115–117 dB) due to the resonance box at room temperature without external heating. The progress of the reaction was monitored by thin-layer chromatography (TLC), and complete conversion was achieved within 10–25 min. Upon completion of the reaction, the precipitated solid was collected by filtration, washed with cold ethanol, and recrystallized from 95% ethanol to afford the pure products (1, 2, 3) in excellent yields.

##### Method B: amplified acoustic activation in the absence of natural quartz nanoparticles

The reaction was performed according to the procedure described in Method A, except that no natural quartz nanoparticles were added. The reaction mixtures were exposed only to amplified sound waves (115–117 dB) at room temperature. Irradiation was interrupted every 30 min for 10–15 min to allow the speaker to cool, and was then continued until the end of the reaction. The reactions required significantly longer times (60–180 min) to reach completion, as confirmed by TLC. The resulting solids were filtered, washed with ethanol, and recrystallized from 95% ethanol following the same procedure described for Method A.

##### Method C: conventional stirring at room temperature

The reactions were carried out using the same reactant ratios described in Method A but in the absence of both natural quartz nanoparticles and amplified sound waves. The reaction mixtures were stirred at room temperature and monitored by TLC. No detectable product formation was observed even after standing overnight, indicating that no reaction occurs under these mild conditions in the absence of both acoustic activation and the quartz nanocatalyst.

###### 2-Hydroxy-4,6-dimethylnicotinonitrile (1)

White solid, m.p.: 362–365 °C, FT-IR (KBr): *ν̄*_max_ 3290–2798 (O*H* stretching), 2215 (C≡N), 1624 (C=N ring) cm^−1^. ^1^H NMR (400 MHz, DMSO-d_6_): *δ* = 2.22 (s, 3*H*, C*H*_3_), 2.30 (s, 3*H*, C*H*_3_), 6.15 (s, 1*H*, C*H* ring), 12.10 (s, 1*H*, O*H*, exchangeable by D_2_O) ppm. ^13^C NMR (101 MHz, DMSO-d_6_): *δ* = 19.35, 21.07, 99.54, 107.95, 116.54, 152.72, 161.93, 161.46 ppm.

###### 2,6-Dihydroxy-4-methylnicotinonitrile (2)

White solid, m.p.: 321–323 °C, FT-IR (KBr): *ν̄*_max_ 3286–2835 (O*H* stretching), 2194 (C≡N), 1585(C=N) cm^−1^. ^1^*H* NMR (400 MHz, DMSO-d_6_): *δ* = 1.98 (s, 3*H*, C*H*_3_), 5.02 (s, 1*H*, C*H* ring), 10.05 (br s, 2*H*, 2O*H*, exchangeable by D_2_O) ppm. ^13^C NMR (101 MHz, DMSO-d_6_): *δ* = 20.84, 77.20, 99.91, 116.72, 154.99, 160.50, 166.36 ppm.

###### 2,4,6-trihydroxynicotinonitrile (3)

White solid, m.p.: 311–314 °C, FT-IR (KBr): *ν̄*_max_ 3202–2804 (O*H* stretching), 2264 (C≡N), 1589(C=N) cm^−1^. ^1^*H* NMR (400 MHz, DMSO-d_6_): *δ* = 6.13 (s, 1*H*, C*H* ring), 10.94, 12.18, 13.48 (br s, 3*H*, 3O*H*, exchangeable by D_2_O) ppm. ^13^C NMR (101 MHz, DMSO-d_6_): *δ* = 81.25, 88.07, 118.58, 165.42, 169.61, 170.54 ppm.

#### General procedure for compounds (4, 5, 6)

Compounds 4–6 were prepared according to the same procedures described for compounds 1–3 (Methods A–C), except that malononitrile was used instead of ethyl cyanoacetate. All other reaction conditions and purification procedures remained unchanged.

##### 2-Amino-4,6-dimethylnicotinonitrile (4)

Yellow crystals, mp: 251–253 °C; IR (KBr) cm^−1^: 3491, 3433 (N*H*_2_), 3001 (C–*H* aromatic), 2850, 2795 (C–*H* aliphatic), 2218 (–C≡N), 1608 (N*H* bending); ^1^H NMR (DMSO-d_6_) *δ* ppm: 2.22 (s, 3*H*, C*H*_3_), 2.30 (s, 3*H*, C*H*_3_), 6.15 (s, 1*H*, pyridine–*H*), 6.70 (s, 2*H*, N*H*_2_, D_2_O exchangeable); ^13^C NMR (DMSO-d_6_) *δ* ppm: 19.34 (aliphatic C*H*_3_ at position 4), 21.07 (aliphatic C*H*_3_ at position 6), 99.53 (pyridine C bearing CN), 116.54 (–C≡N), 107.97, 151.71, 160.69, 161.46 (pyridine carbons).

##### 2-Amino-4-hydroxynicotinonitrile (5)

Brown crystals, mp: 166–168 °C; IR (KBr) cm^−1^: 3444 (O*H*), 3367 (N*H*_2_), 3101 (C–*H* aromatic), 2218 (–C≡N); ^1^*H* NMR (DMSO-d_6_) *δ* ppm: 6.46 (s, 2*H*, N*H*_2_), 7.48–7.54 (dd, 2*H*, pyridine–*H*), 10.67 (s, *H*, O*H*); ^13^C NMR (DMSO-d_6_) *δ* ppm: 61.27, 112.37, 116.61, 148.70, 166.98, 174.90.

##### 2-Amino-4-methylnicotinonitrile (6)

Yellow crystals, mp: 150–151 °C; IR (KBr) cm^−1^: 3367–3330 (N*H*_2_), 3102 (C–*H* aromatic), 2218 (–C≡N); ^1^*H* NMR (DMSO-d_6_) *δ* ppm: 2.20 (s, 3*H*, C*H*_3_), 6.96 (s, 2*H*, N*H*_2_, D_2_O exchangeable), 7.80–7.86 (dd, 2*H*, pyridine–*H*); ^13^C NMR (DMSO-d_6_) *δ* ppm: 24.62, 84.11, 110.12, 116.72, 157.86, 160.56, 162.92.

##### 4,6-Diamino-2-hydroxynicotinonitrile (7)

Compound 7 were prepared according to the same procedures described for compounds 1–3 (Methods A–C), except that malononitrile was used instead of ethyl cyanoacetate which reacts with other mole of ethyl cyanoacetate or malononitrile. All other reaction conditions and purification procedures remained unchanged. Yellow crystals, mp: 180–182 °C; IR (KBr) cm^−1^: 3421 (O*H*), 3348–3310 (N*H*_2_), 3197 (C–*H* aromatic), 2189 (–C≡N); ^1^*H* NMR (DMSO-d_6_) *δ* ppm: 4.34 (s, 2*H*, N*H*_2_, D_2_O exchangeable), 7.31 (s, 2*H*, N*H*_2_, D_2_O exchangeable), 7.85 (s, 1*H*, pyridine–*H*), 12.24 (s, *H*, O*H*, D_2_O exchangeable); ^13^C NMR (DMSO-d_6_) *δ* ppm: 61.35, 70.45, 116.55, 154.19, 165.57, 167.41.

## Conclusion

Natural quartz nanoparticles (NQNs) were employed as a recyclable catalyst for the synthesis of pyridine derivatives under ambient conditions in an amplified acoustic field generated with a granite chamber. The combination markedly shortened the reaction times (four- to twelve-fold relative to acoustic irradiation alone, and by more than 90% relative to the reported thermal protocol for compounds 1 and 2) and gave high isolated yields, with an estimated electrical energy input lower than that of the thermal benchmark. A piezoelectric contribution of quartz (*d*_33_ = 3.3 ± 0.2 pC/N for the compacted powder) is proposed as an additional activation factor but has not been demonstrated: controls that separate piezoelectric effects from silanol-based, mixing and thermal effects, kinetic and spectroscopic evidence, and scale-up remain to be addressed. The reaction-step mass metrics (PMI, *E*-factor) are higher than those of the aqueous thermal benchmark because of the solvent volume used. Because respirable quartz nanoparticles are hazardous, appropriate safety precautions are essential.

## Author contributions

H. A. Morsy conceived the research idea and supervised the overall study. Hassan A. El-Sayed carried out the synthesis of the compounds and performed the spectral characterization. Mohamed G. Assy & Doaa A. Elsayed assisted in data analysis, manuscript preparation, and literature review. All authors discussed the results, contributed to the final version of the manuscript, and approved the submitted version.

## Conflicts of interest

The authors declare that there are no known financial or personal conflicts of interest that could have influenced the outcome of this research.

## Supplementary Material

RA-016-D6RA06595C-s001

## Data Availability

All data generated or analyzed in this study are included in this published article and its supplementary information (SI). Supplementary information: spectra and environmental and health safety guidelines for quartz nanoparticles. See DOI: https://doi.org/10.1039/d6ra06595c.
