# Supplementary material for: Electroacoustic activation of natural quartz nanoparticles for the Guareschi–Thorpe synthesis of novel pyridine derivatives
Source: RSC Adv. 2026 Oct 15;16(47):63198–213. doi: 10.1039/d6ra06595c (PMC13671111; doi:10.1039/d6ra06595c)
Supplement: Supplementary file 1 [file RA-016-D6RA06595C-s001.pdf]

Supplementary Information (SI) for RSC Advances.
This journal is © The Royal Society of Chemistry

SPECTRAL ANALYSIS

2-hydroxy-4,6-dimethylnicotinonitrile (1)

IR Spectrum of (1):

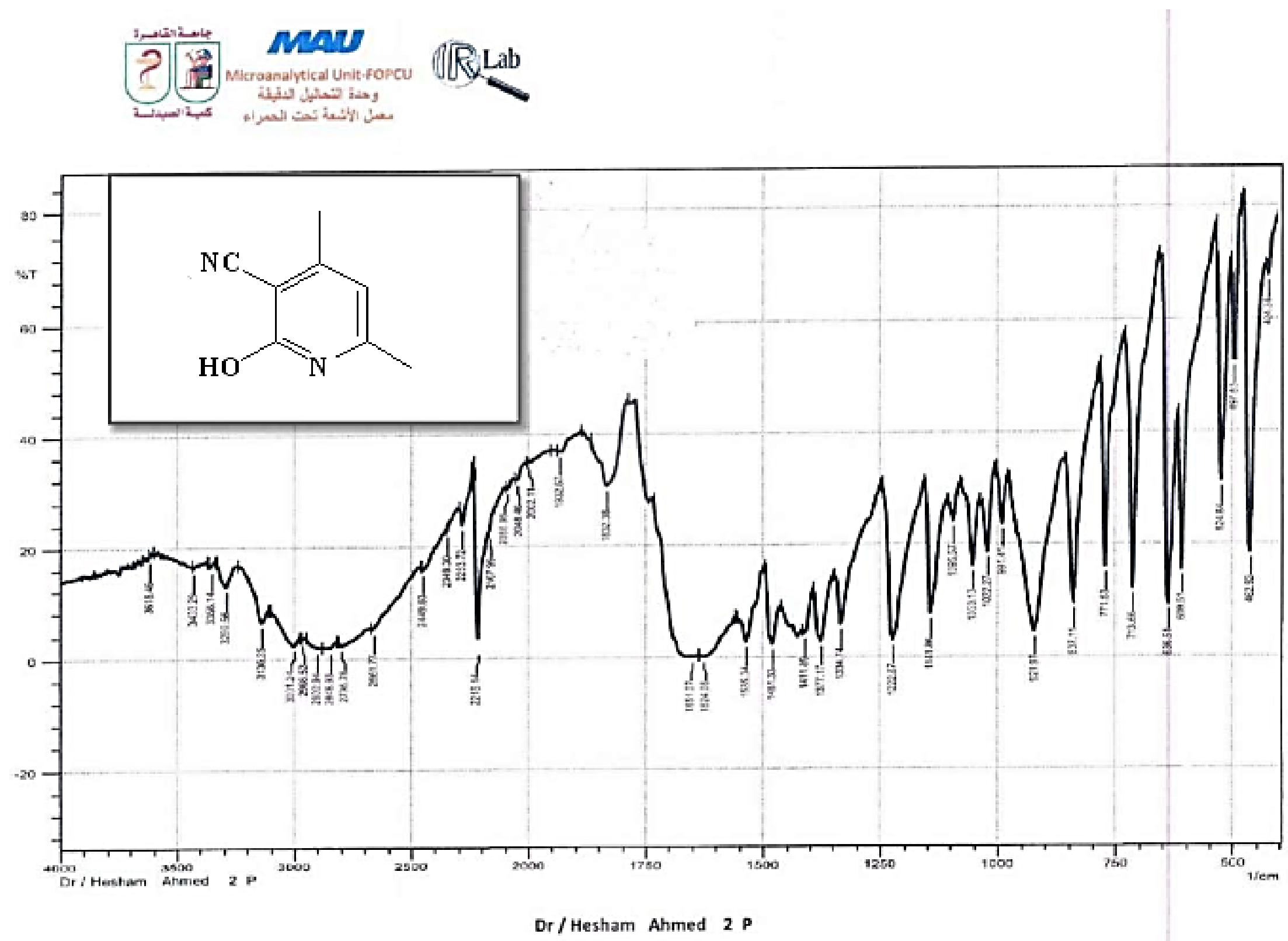

^{1}H-NMR Spectrum of (1):

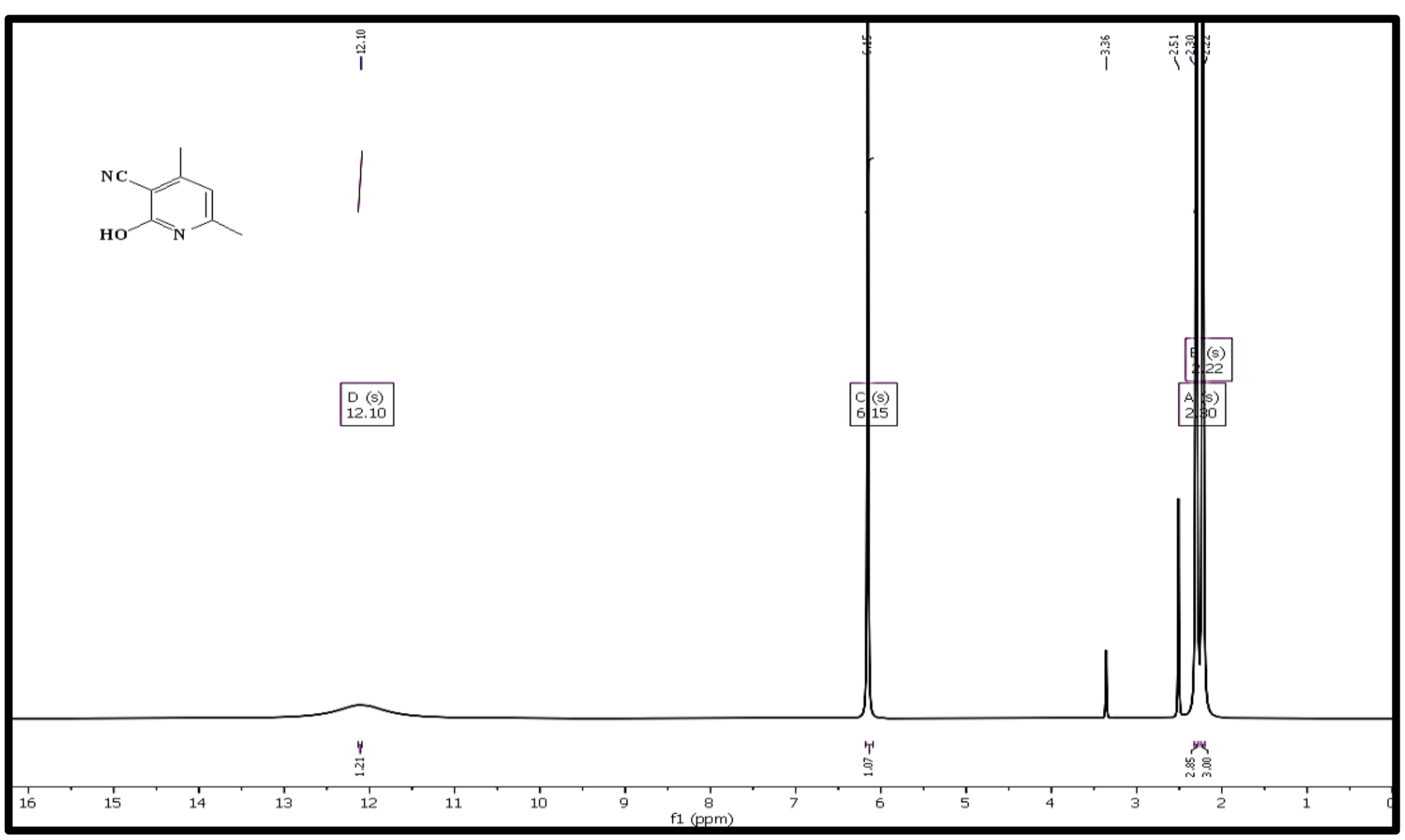

D_2O Spectrum of (1):

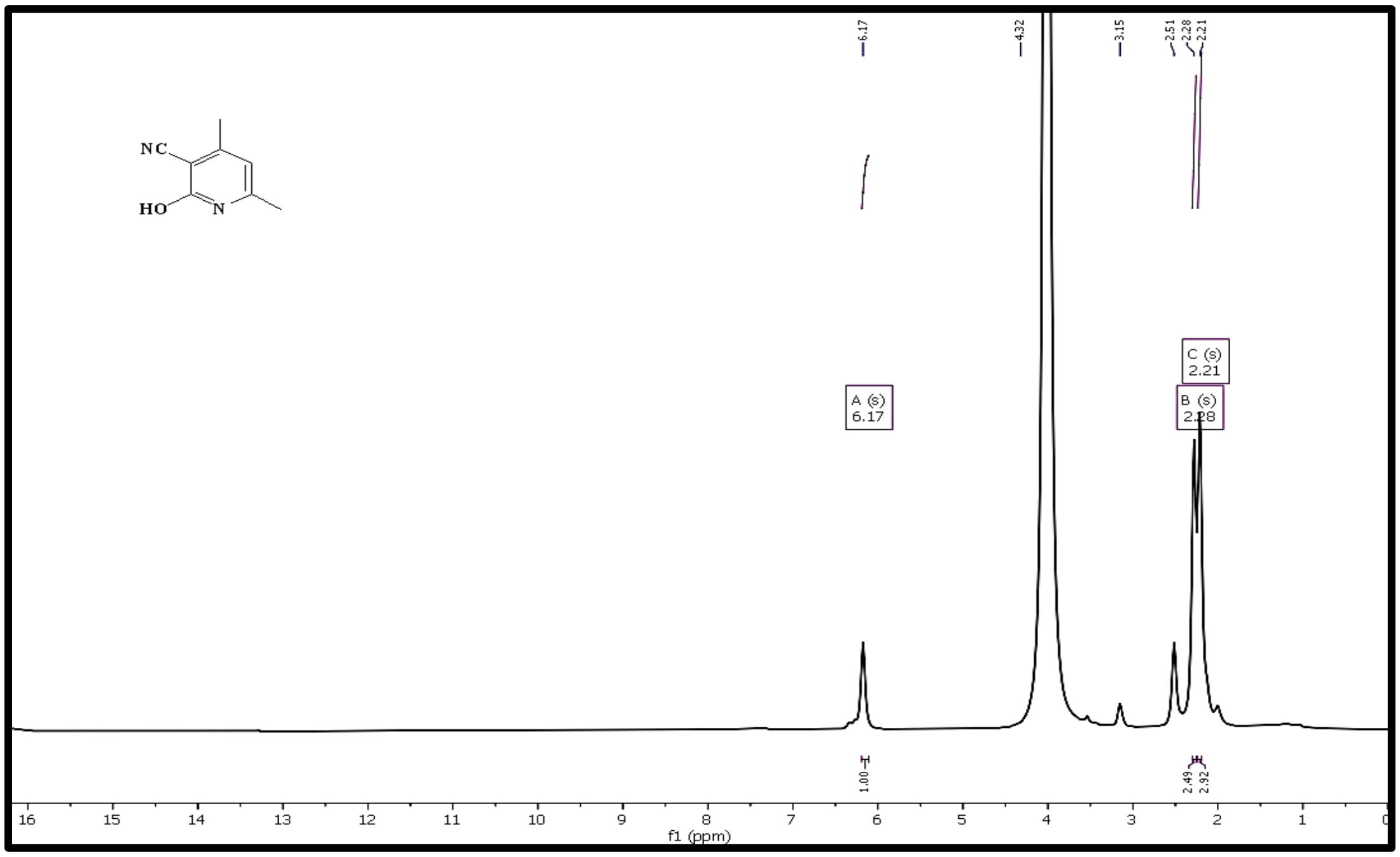

^{13}C-NMR Spectrum of (1):

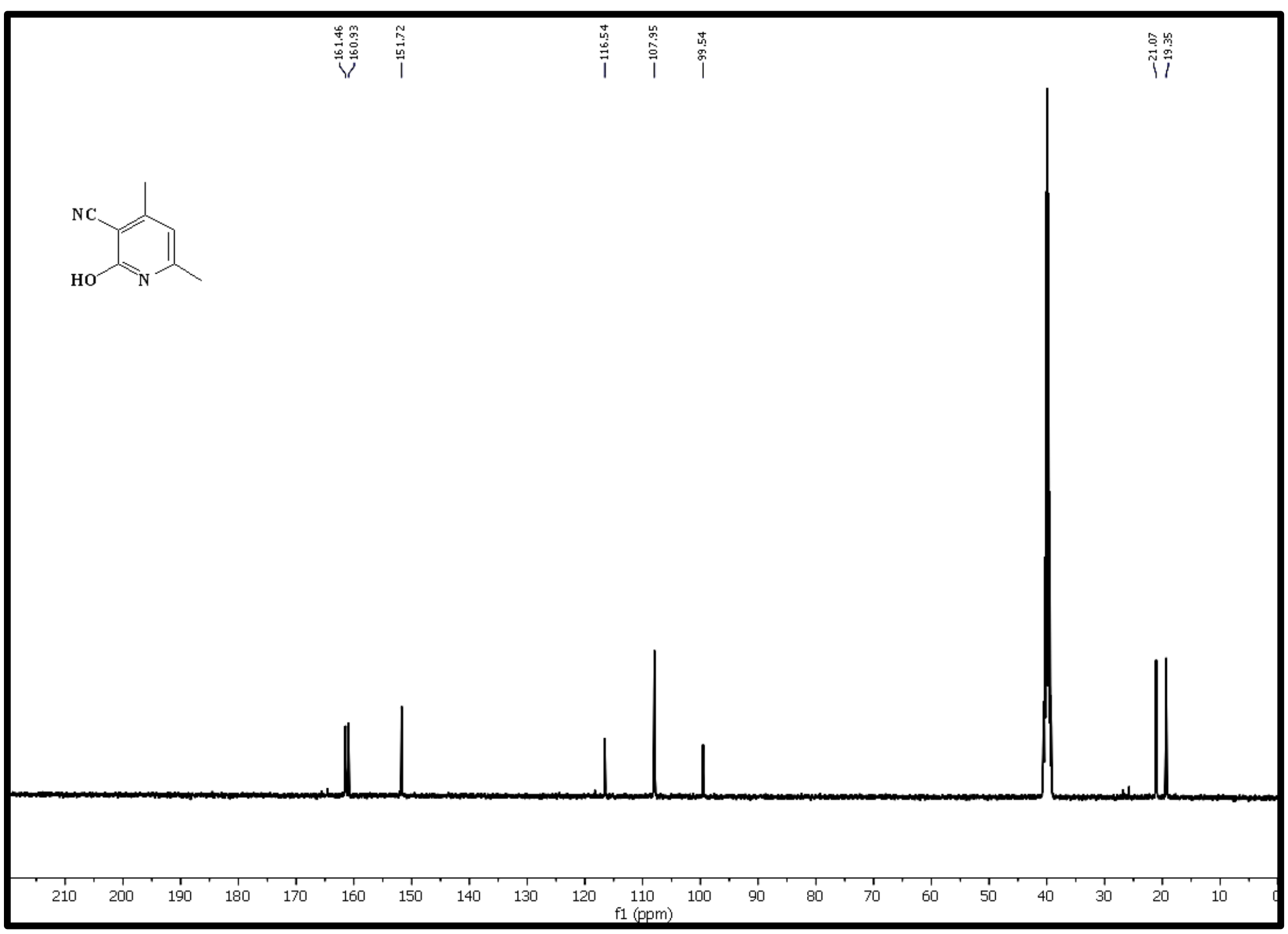

2,6-dihydroxy-4-methylnicotinonitrile (2)

IR Spectrum of (2):

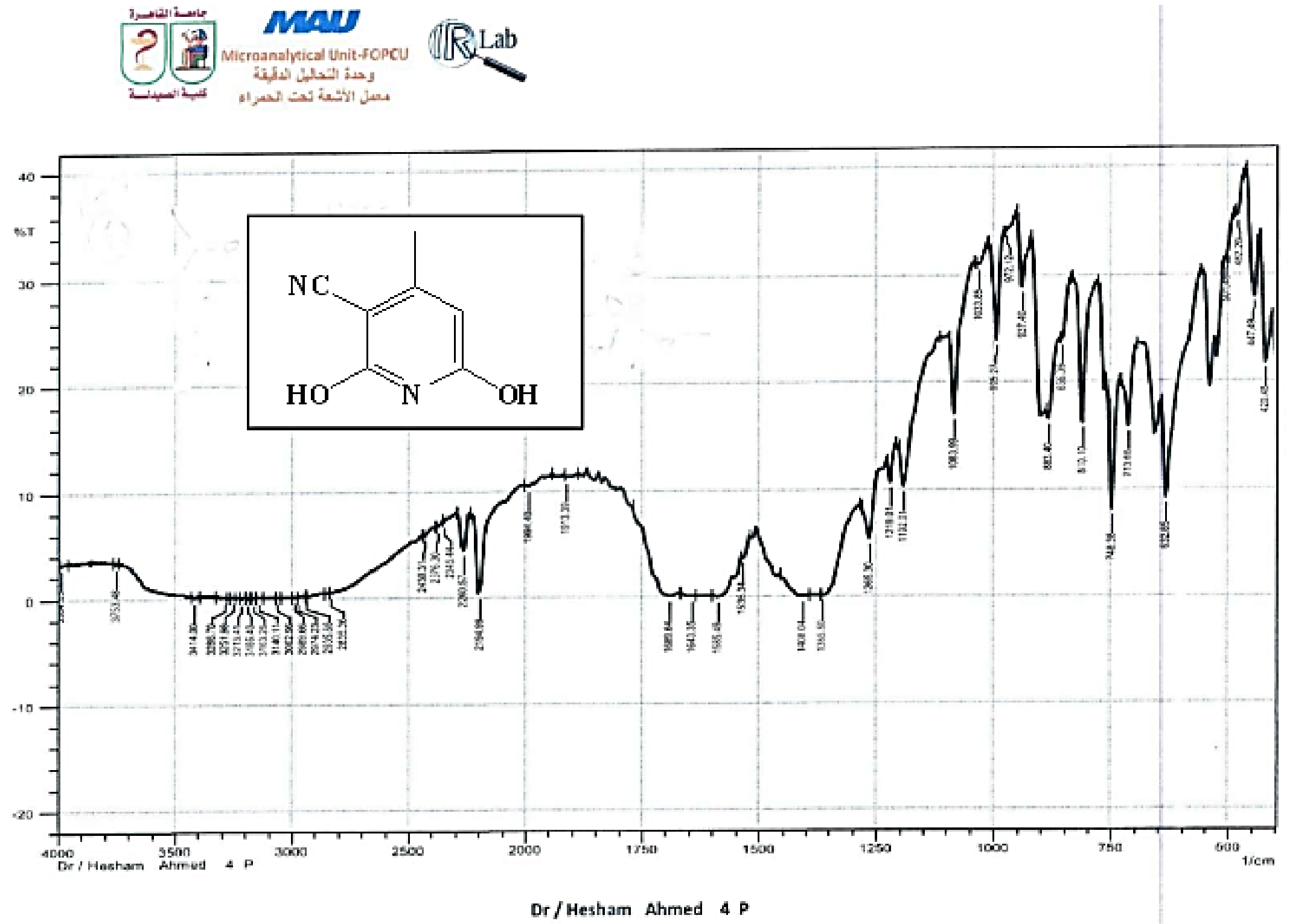

¹H-NMR Spectrum of (2):

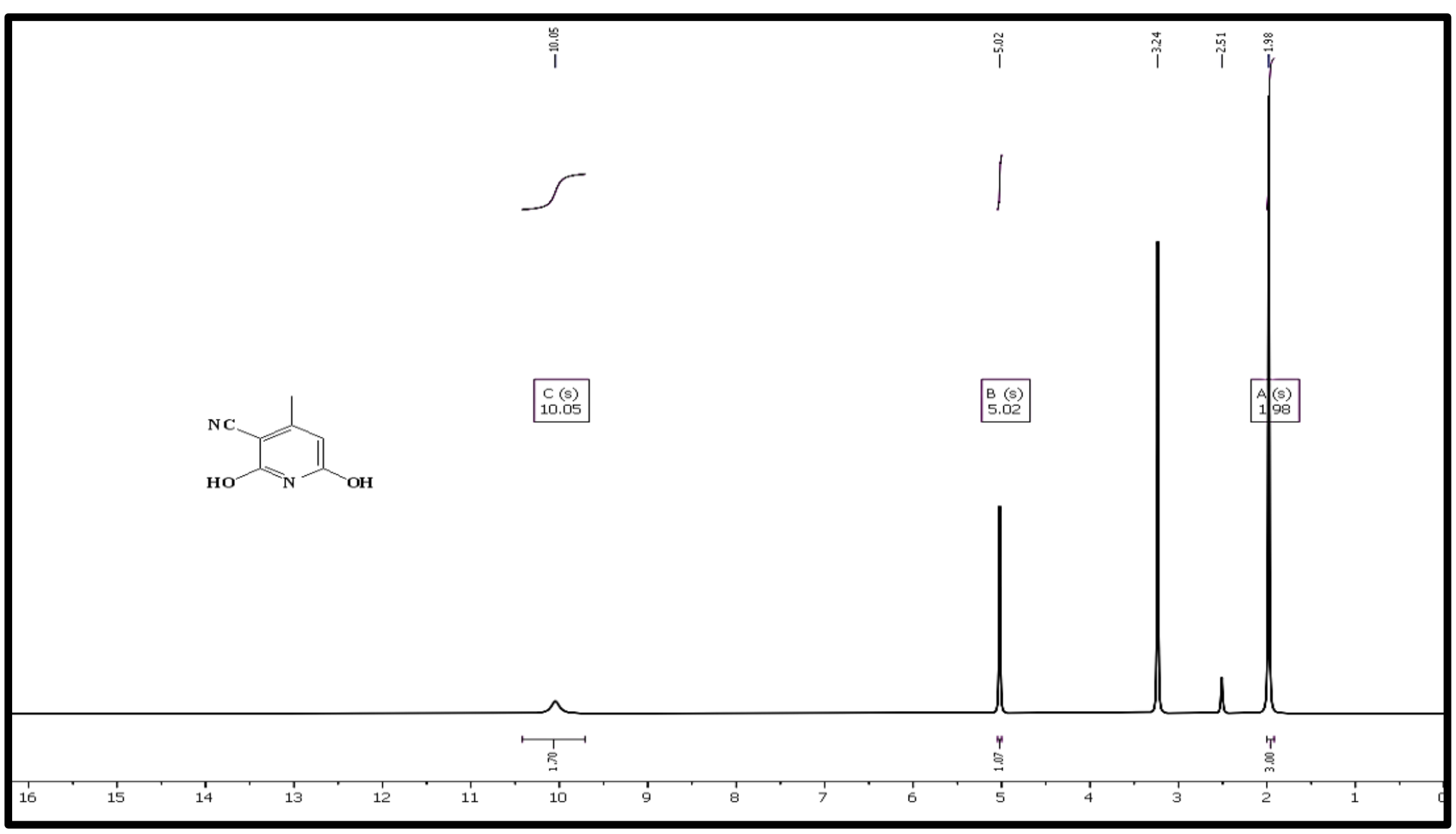

D_2O Spectrum of (2):

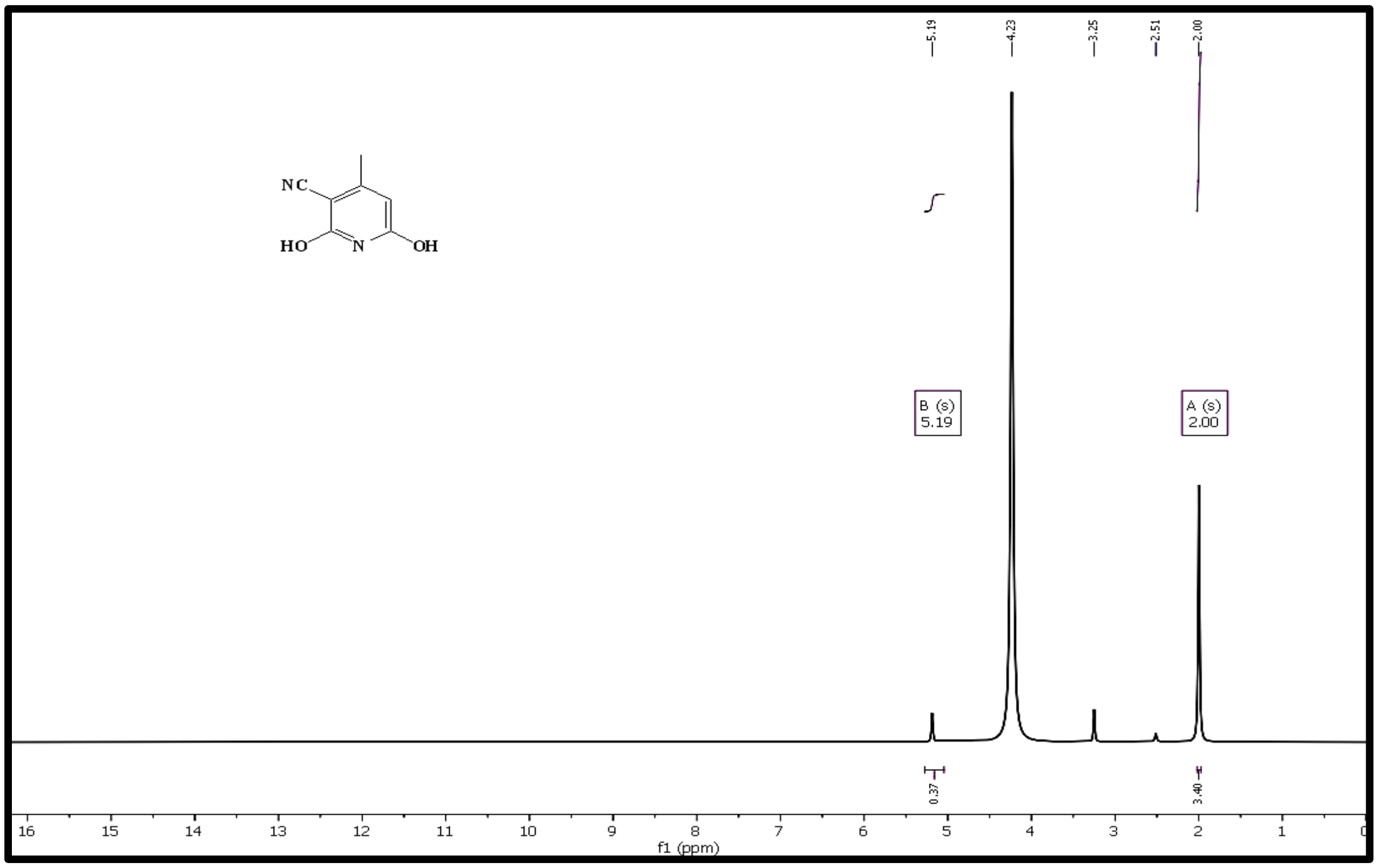

^{13}C-NMR Spectrum of (2):

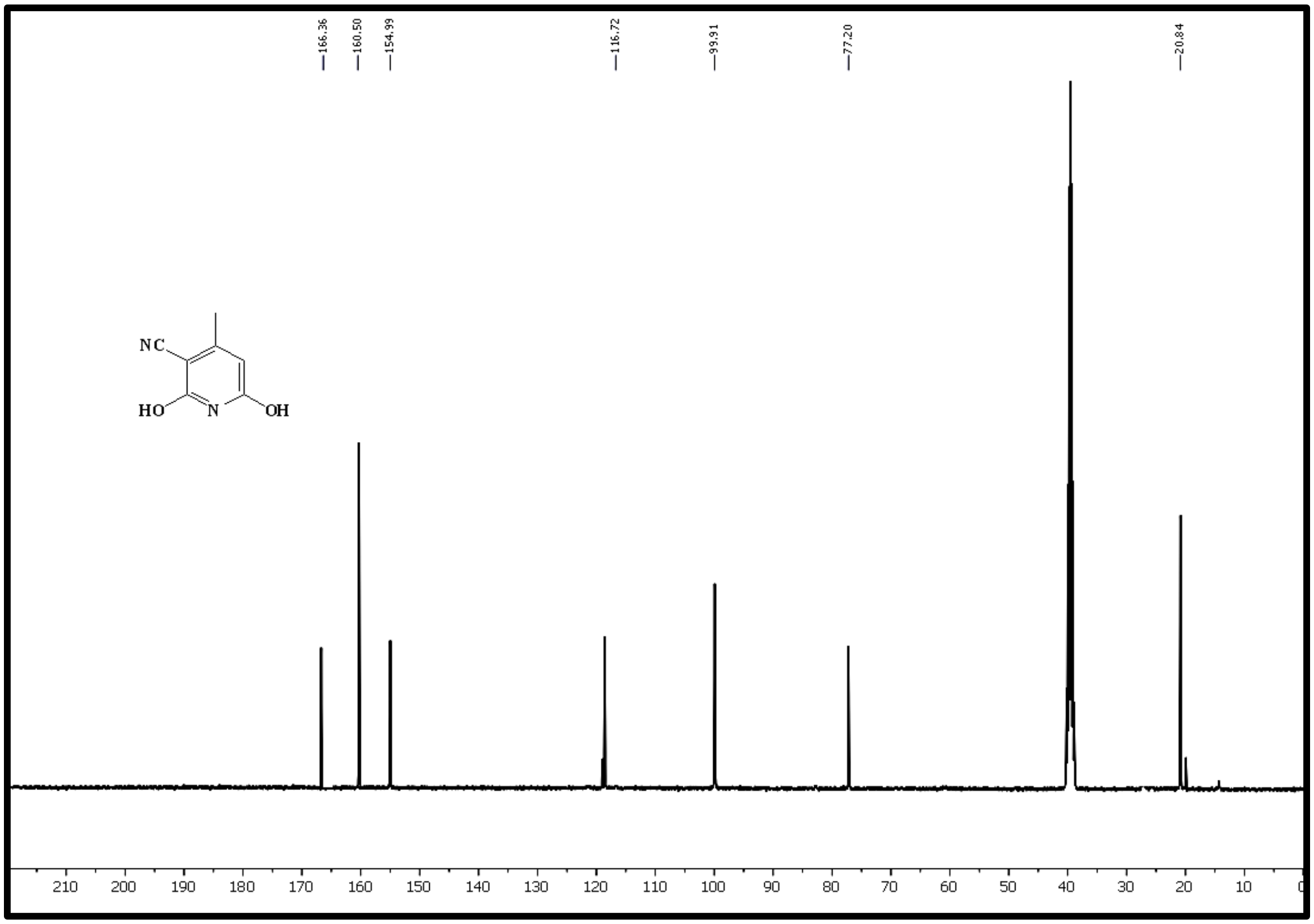

2,4,6-trihydroxynicotinonitrile (3)

IR Spectrum of (3):

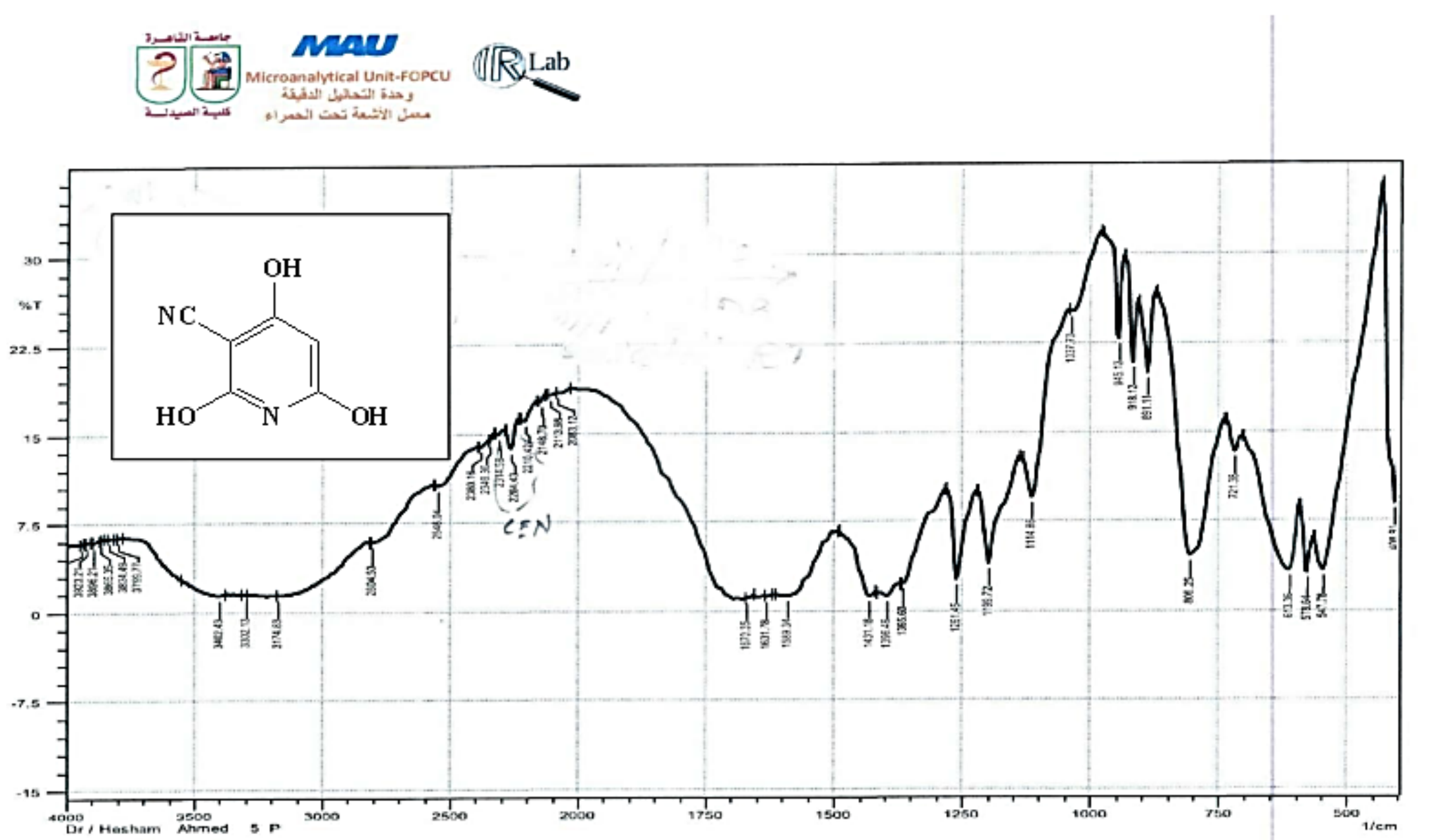

^{1}H-NMR Spectrum of (3):

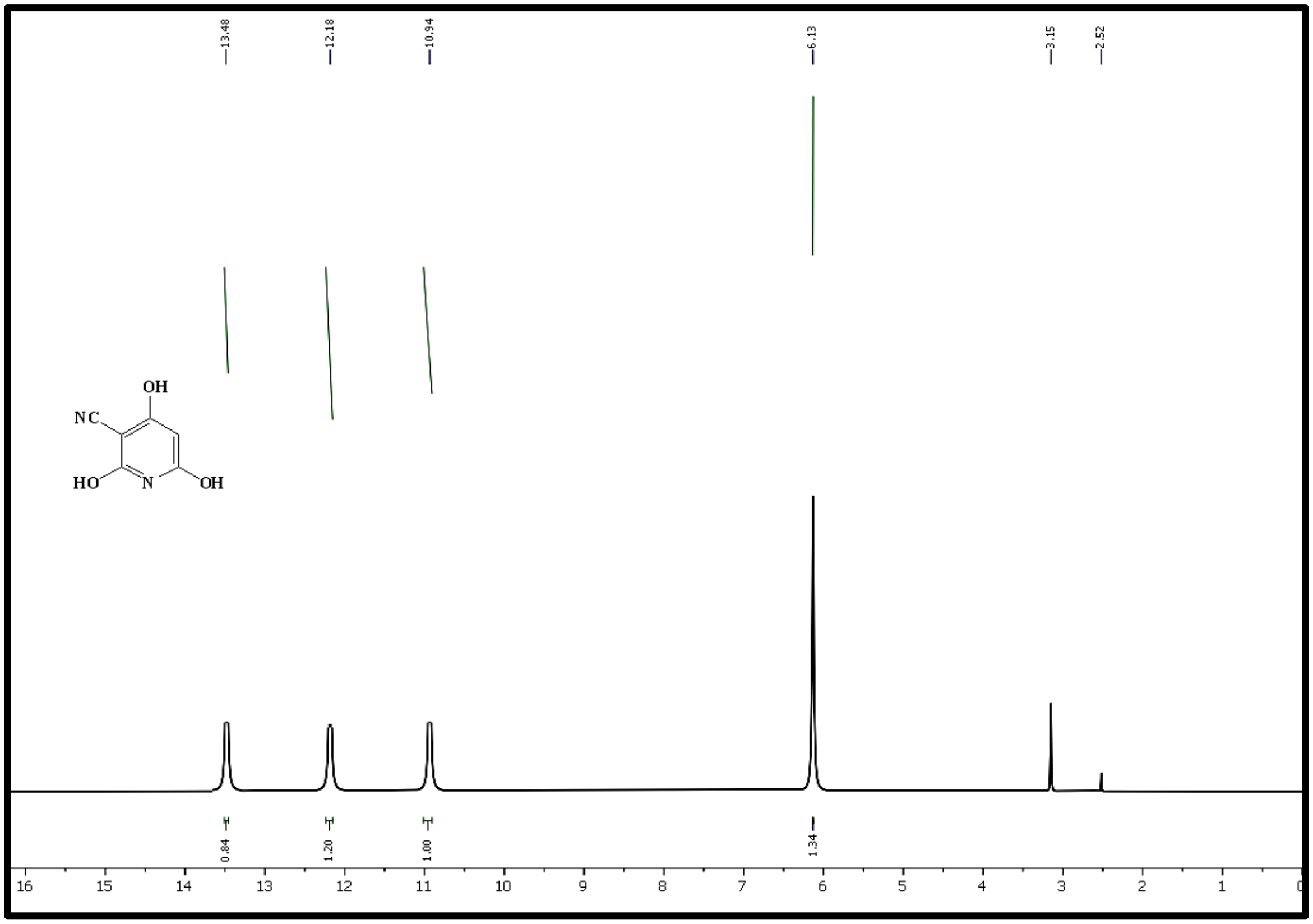

D_2O Spectrum of (3):

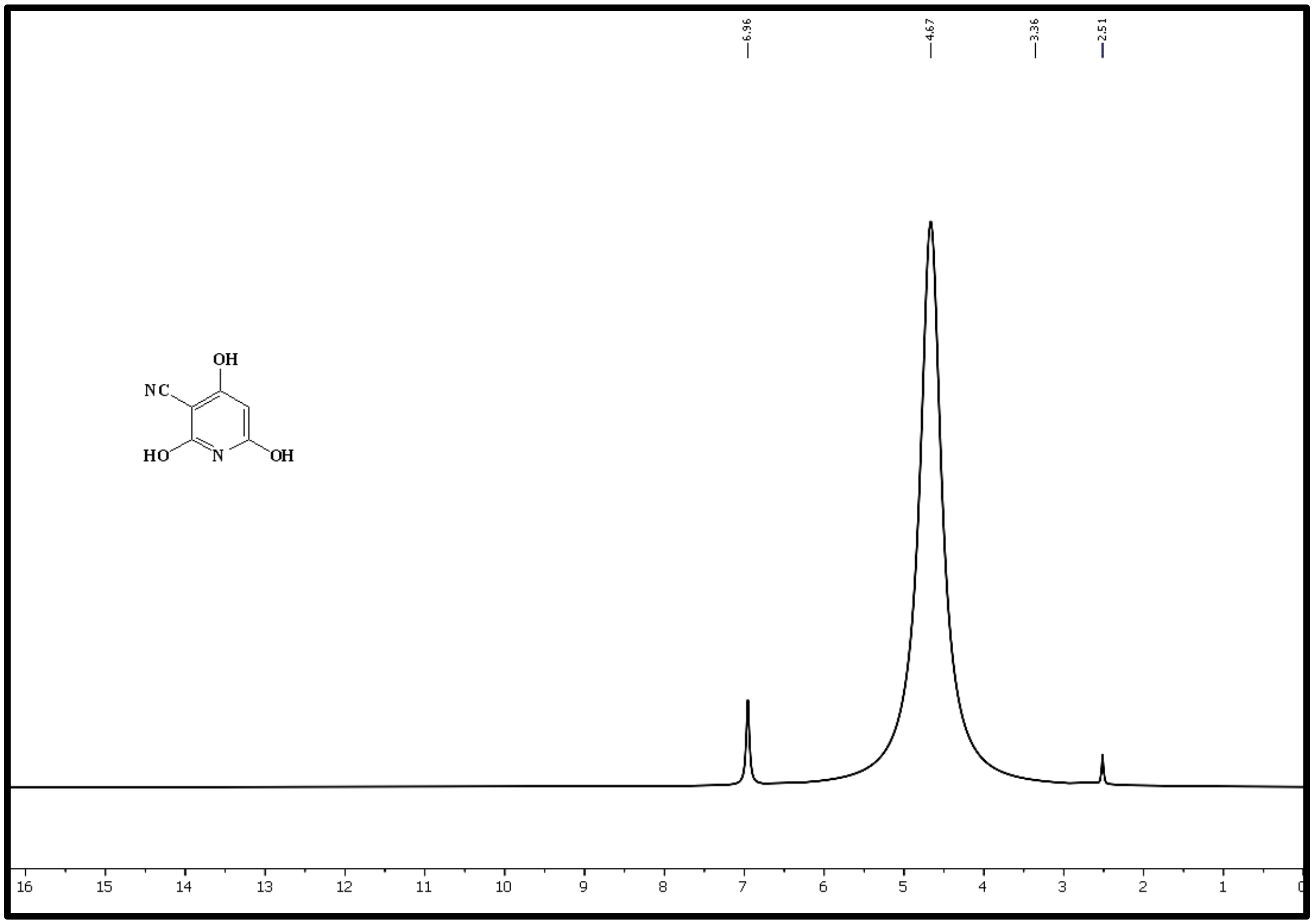

^{13}C-NMR Spectrum of (3):

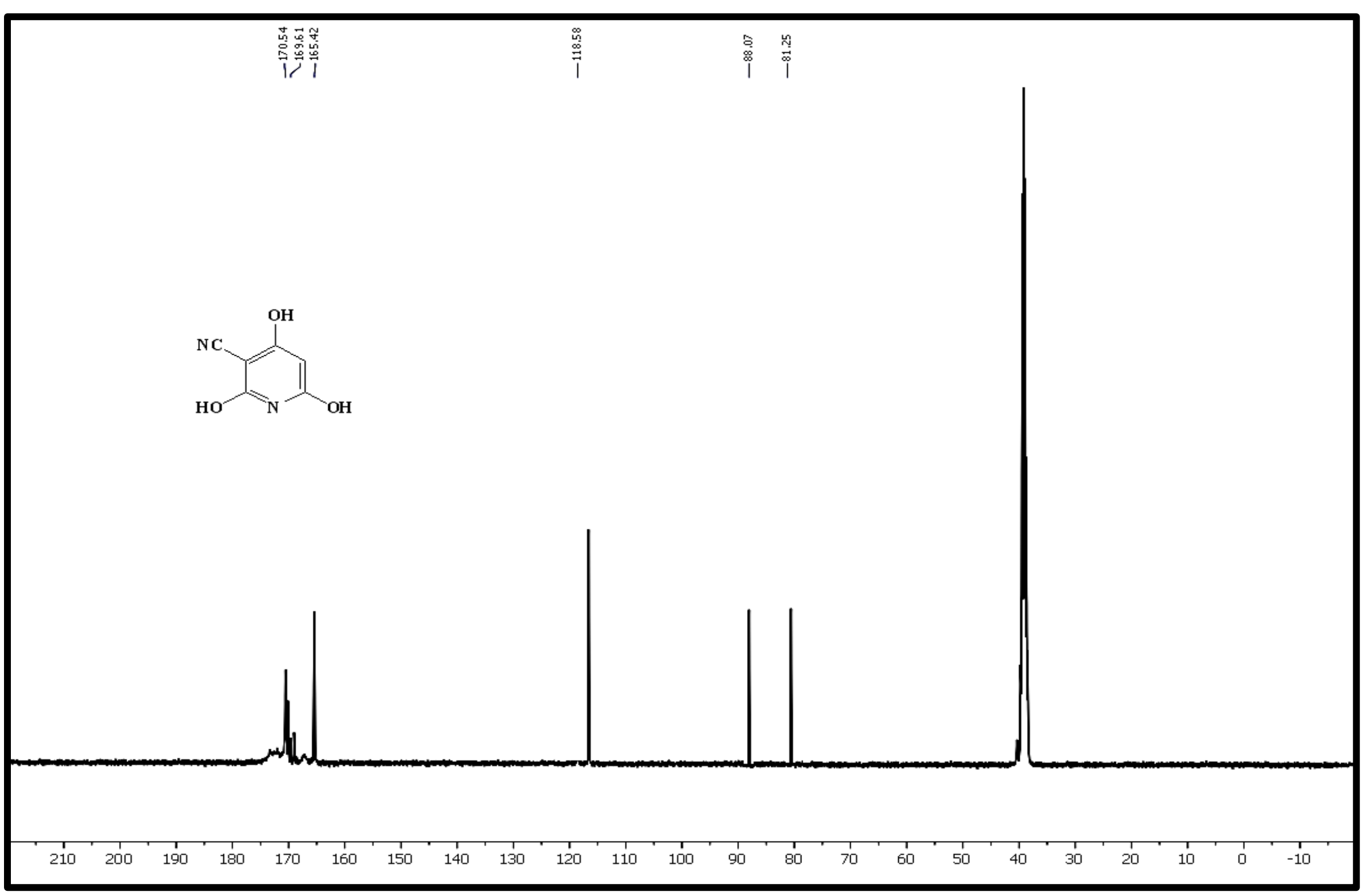

2-amino-4,6-dimethylnicotinonitrile (4)

IR Spectrum of (4):

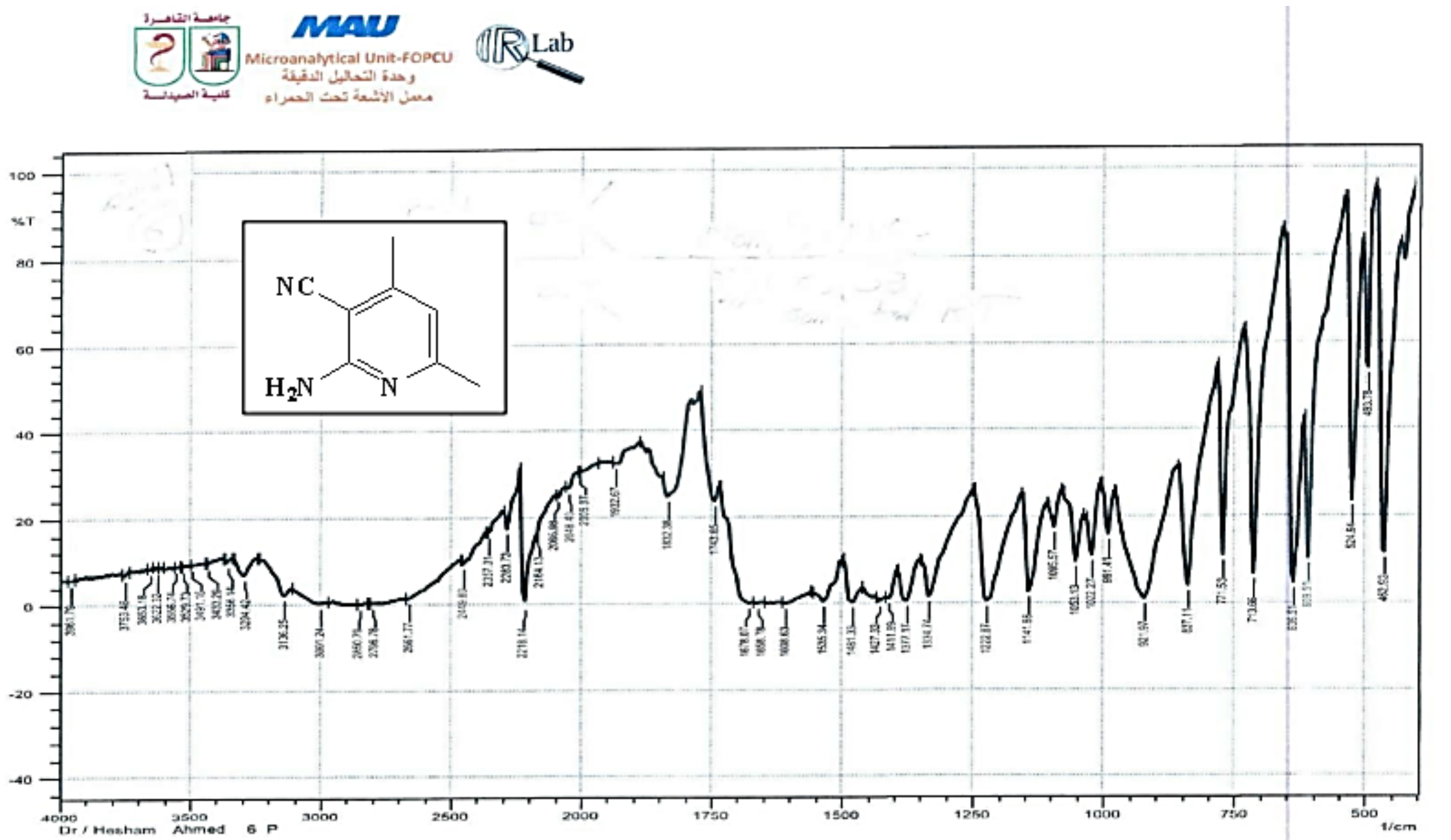

^{1}H-NMR Spectrum of (4):

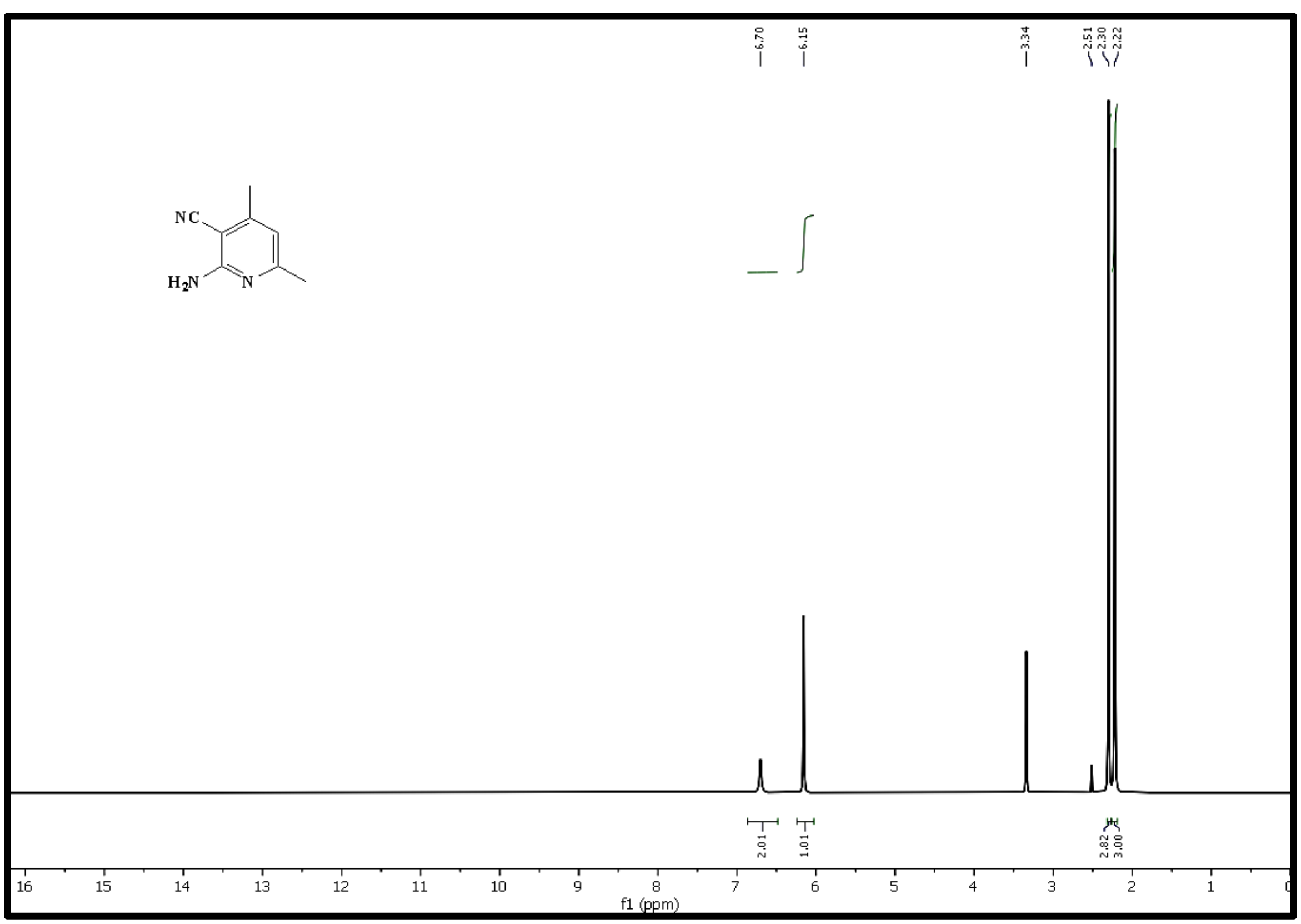

D_2O Spectrum of (4):

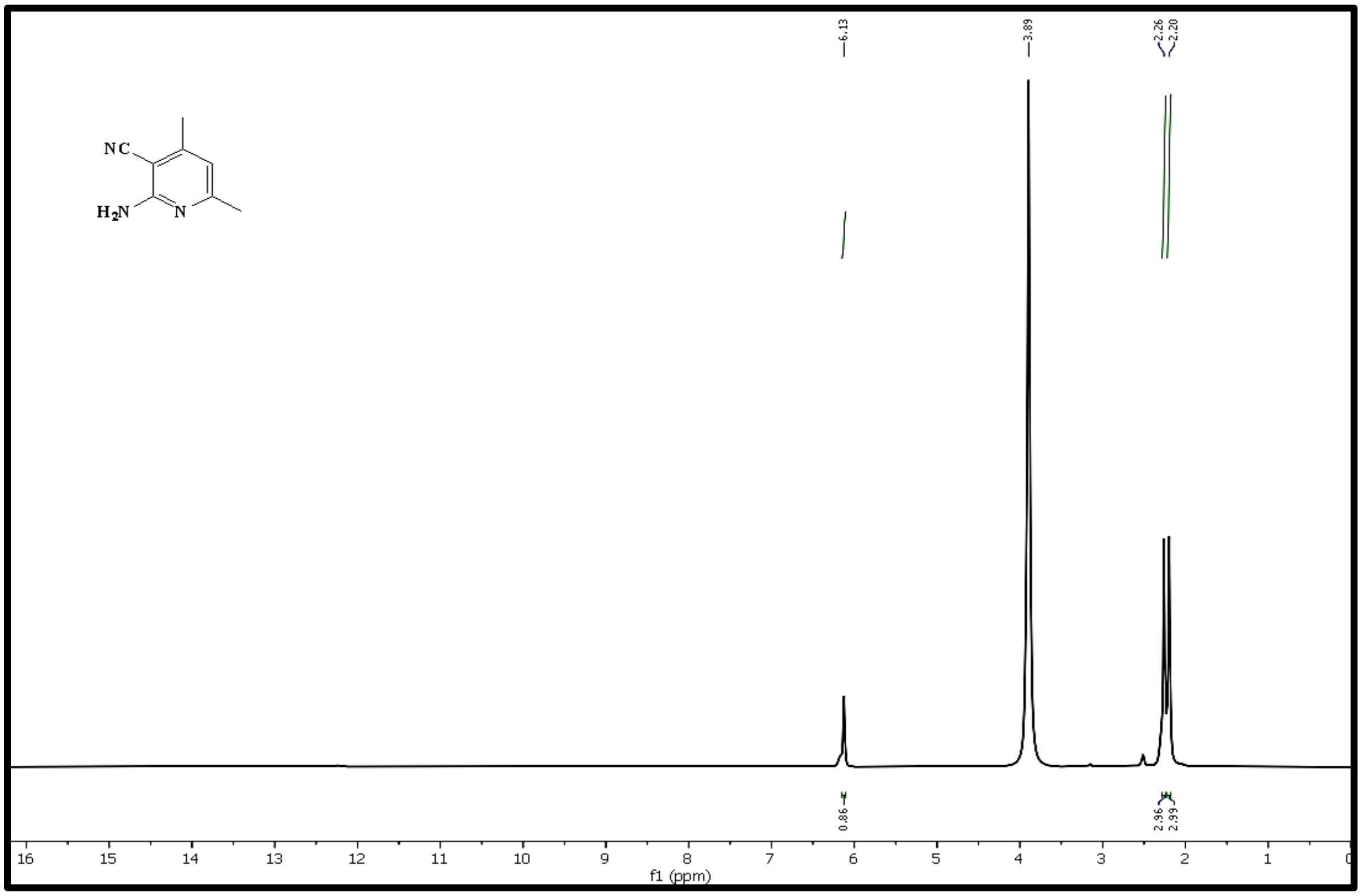

^{13}C-NMR Spectrum of (4):

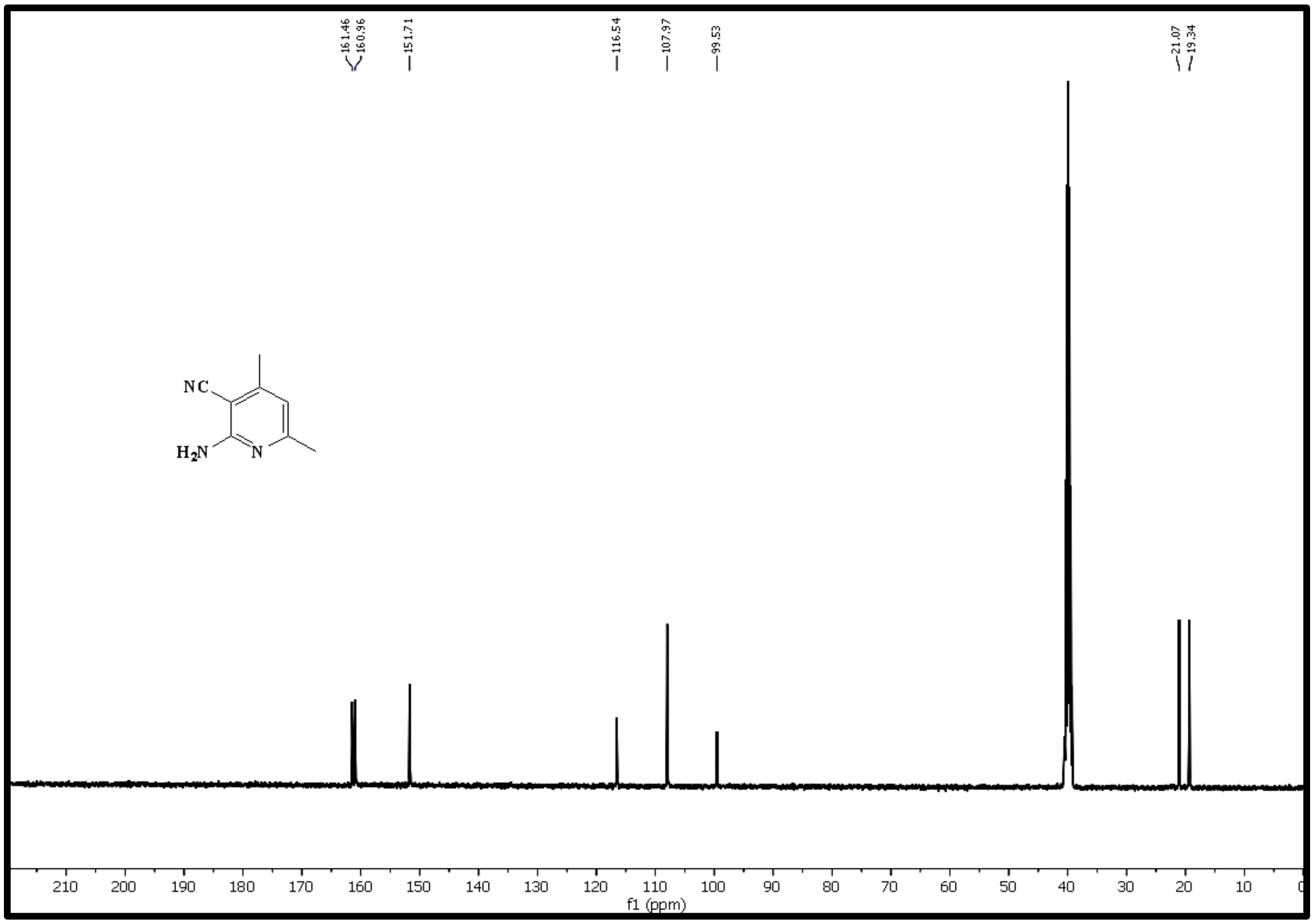

2-amino-4-hydroxynicotinonitrile (5)

IR Spectrum of (5):

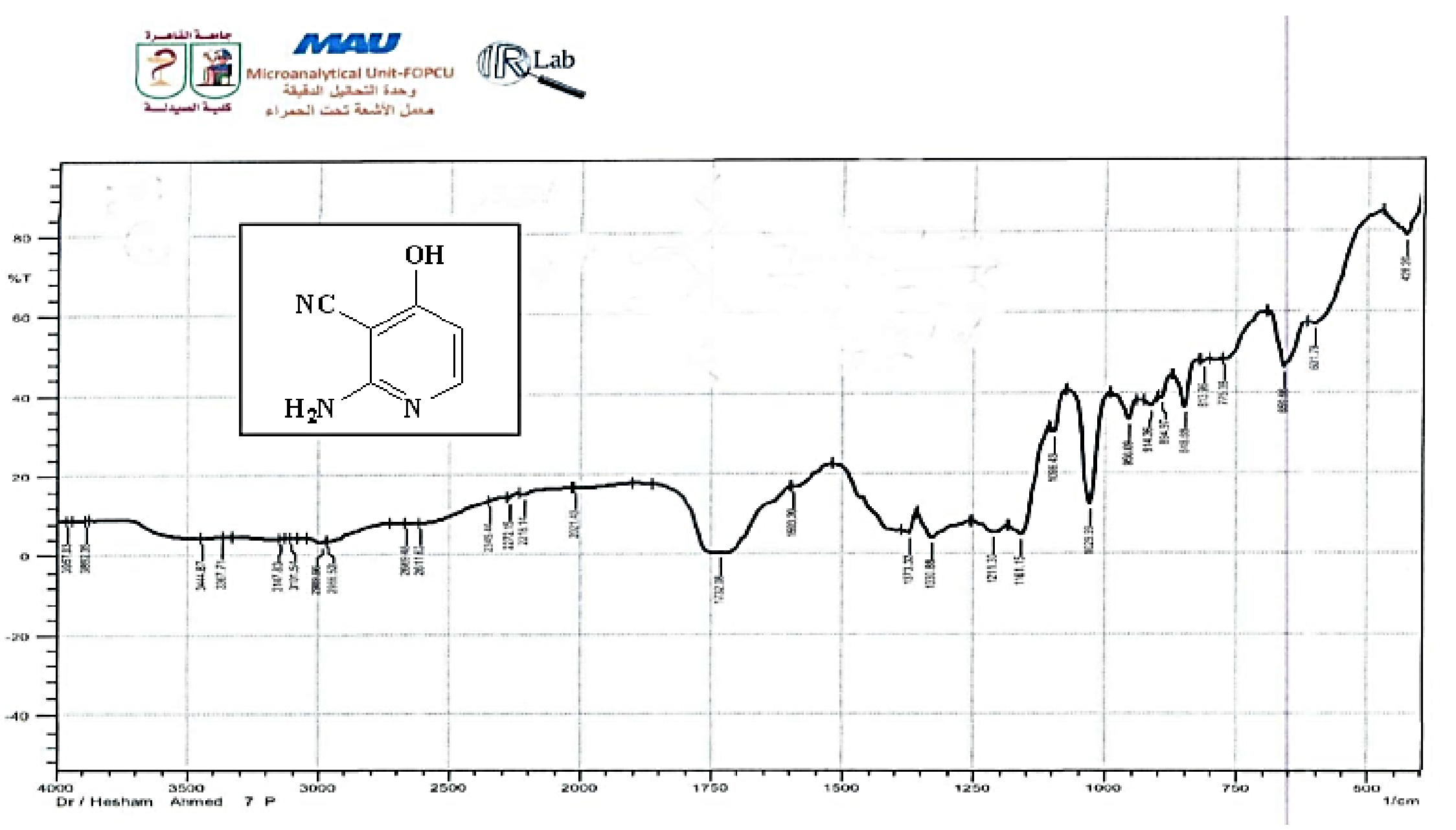

^{1}H-NMR Spectrum of (5):

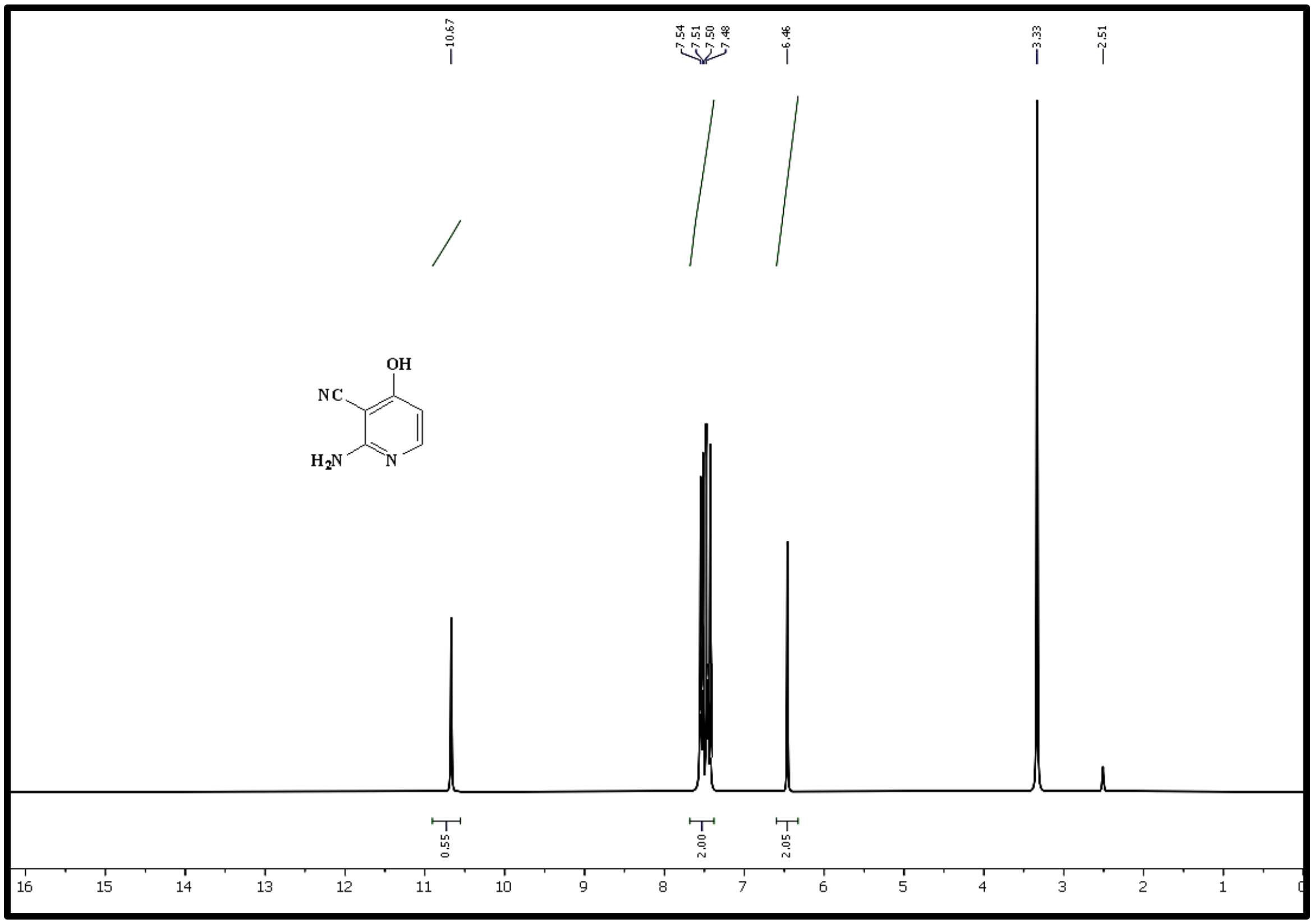

^{13}C-NMR Spectrum of (5):

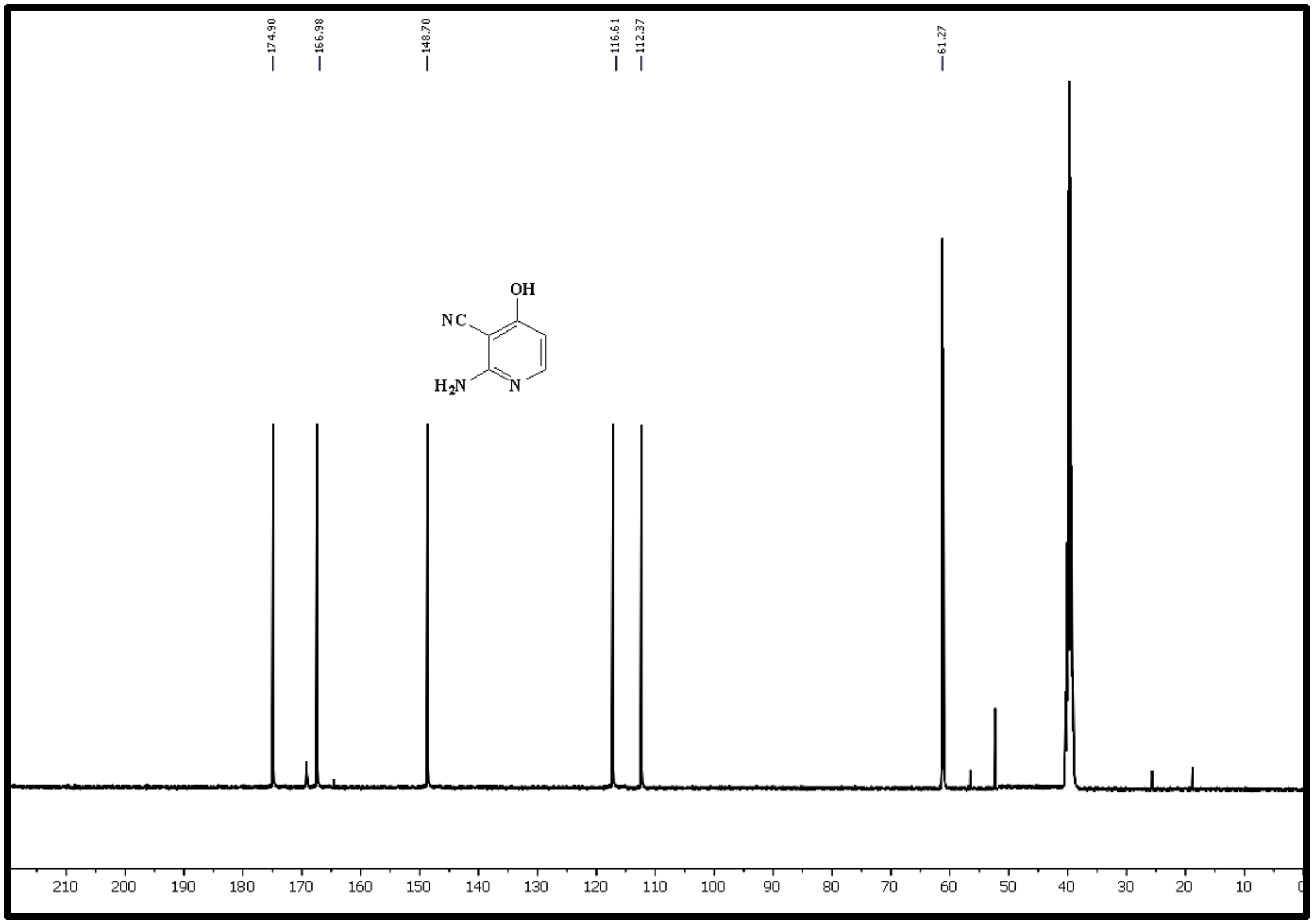

2-amino-4-methylnicotinonitrile (6)

IR Spectrum of (6):

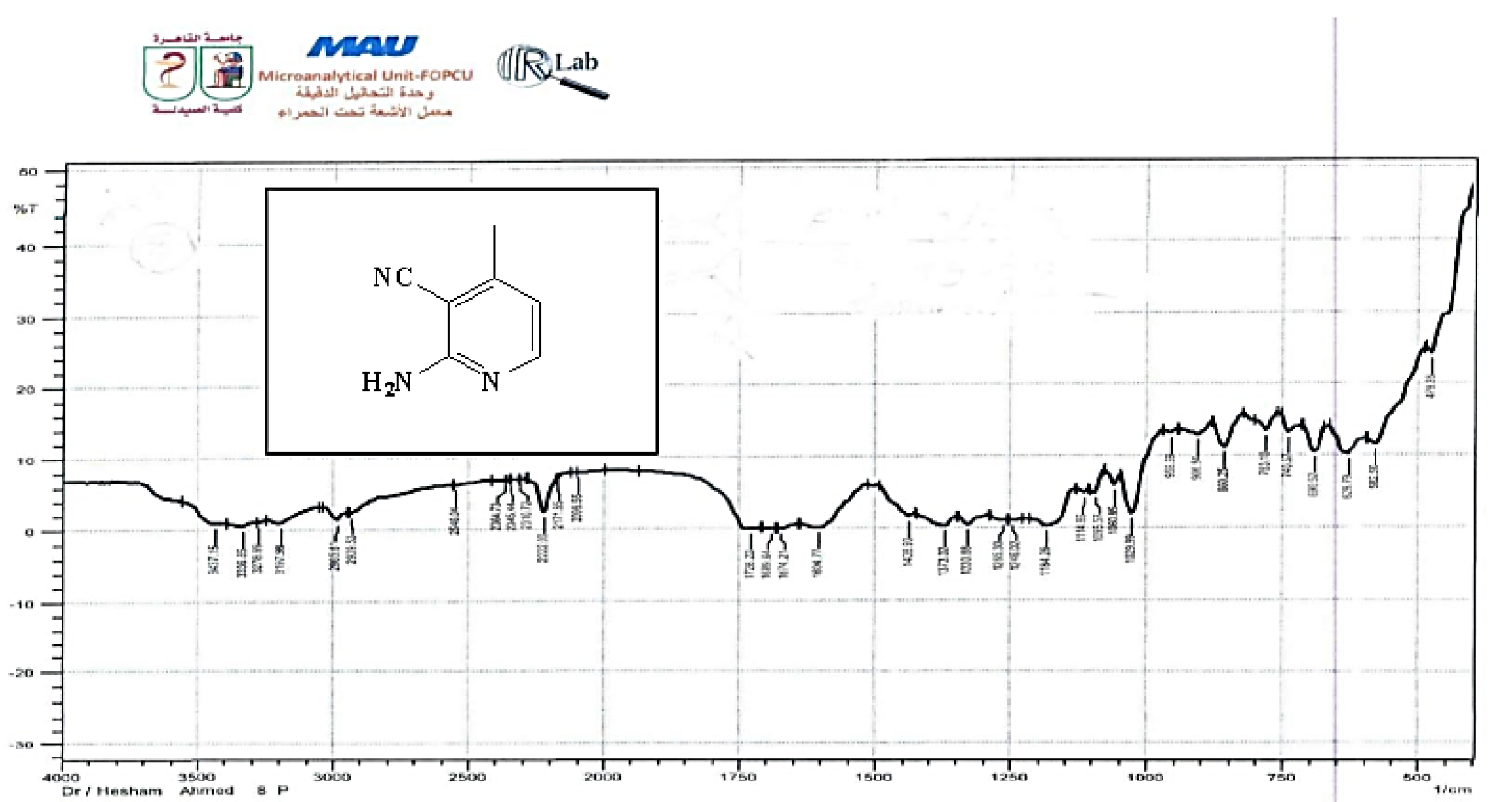

^{1}H-NMR Spectrum of (6):

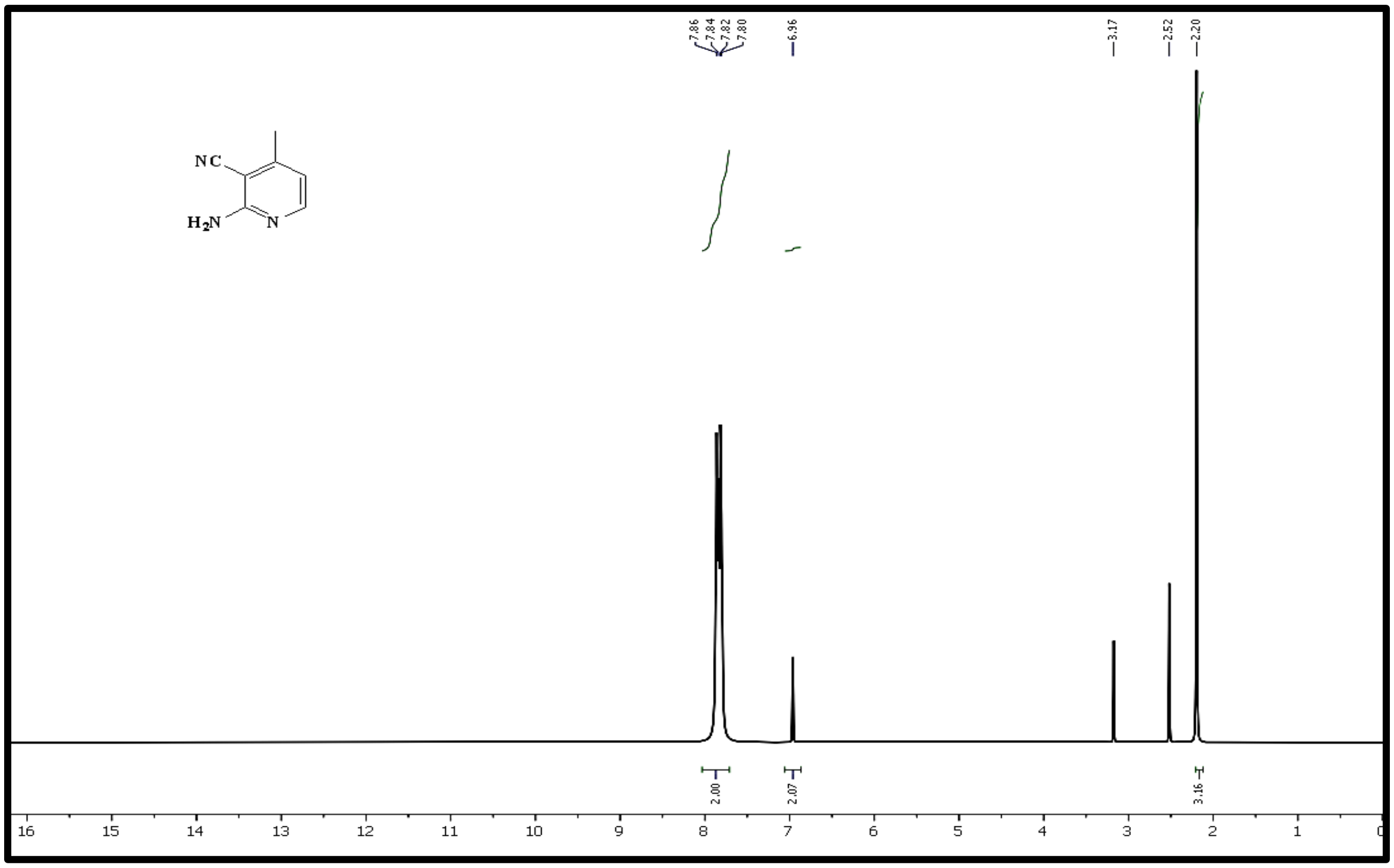

D_2O Spectrum of (6):

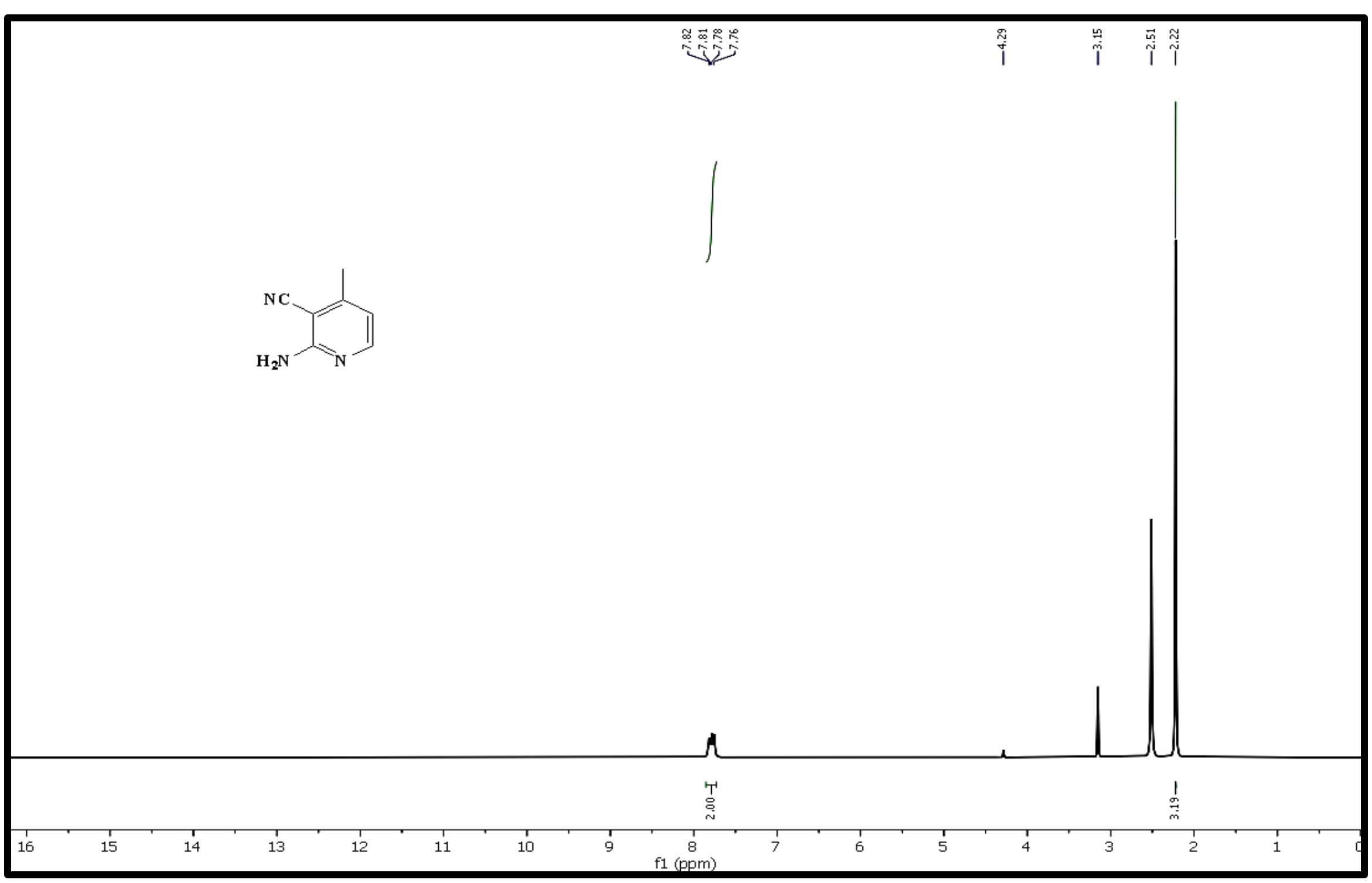

^{13}C-NMR Spectrum of (6):

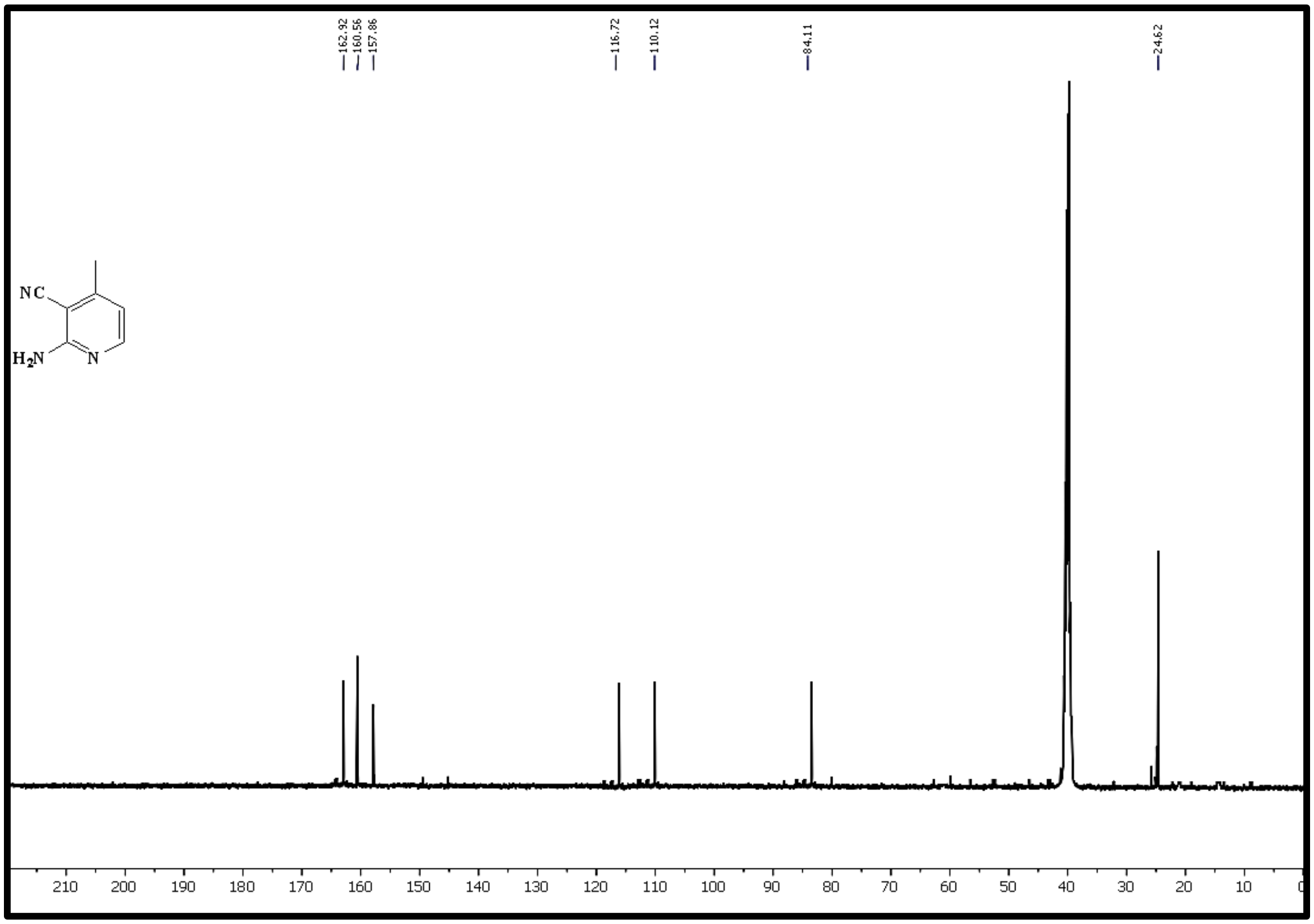

4,6-diamino-2-hydroxynicotinonitrile (7)

IR Spectrum of (7):

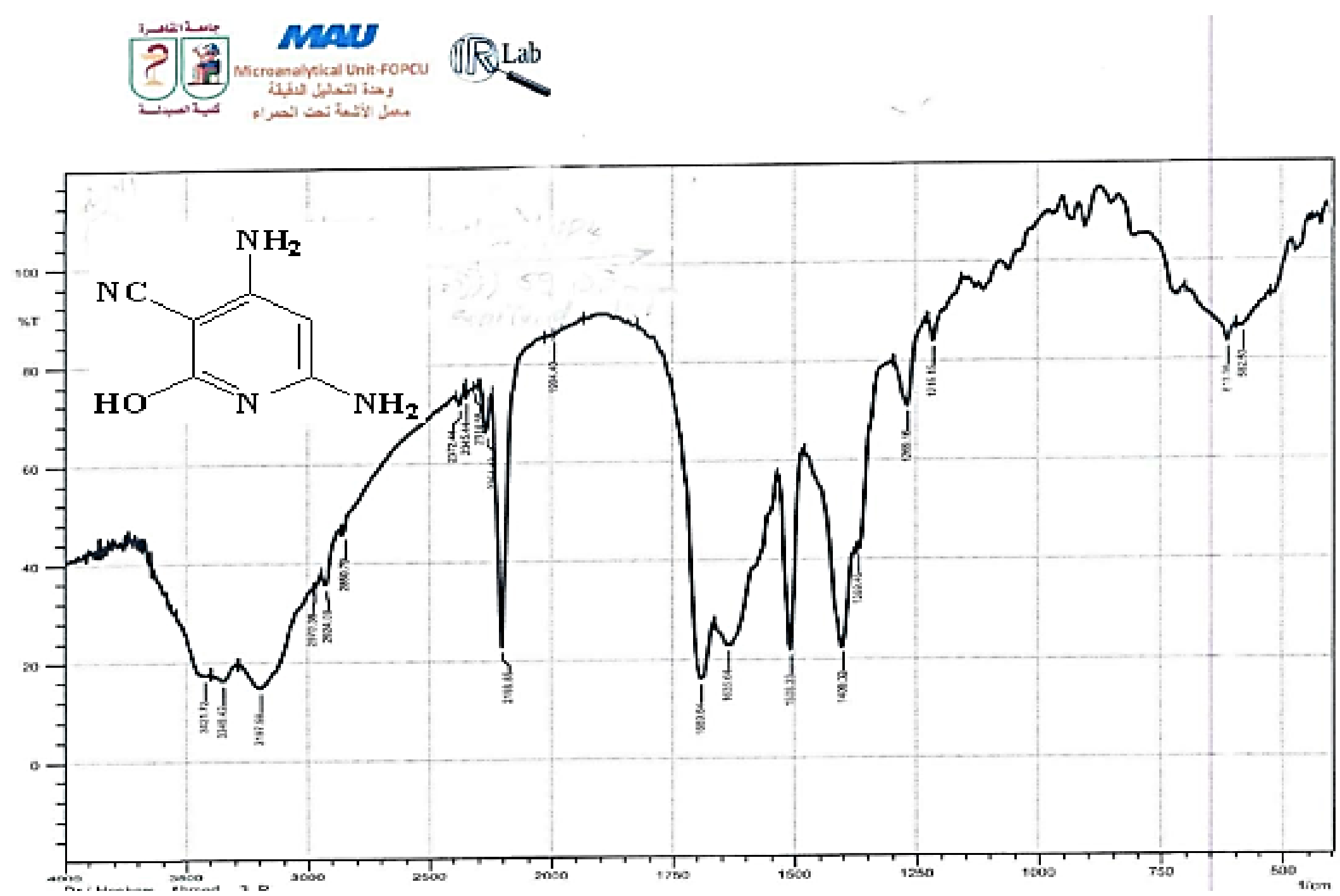

^{1}H-NMR Spectrum of (7):

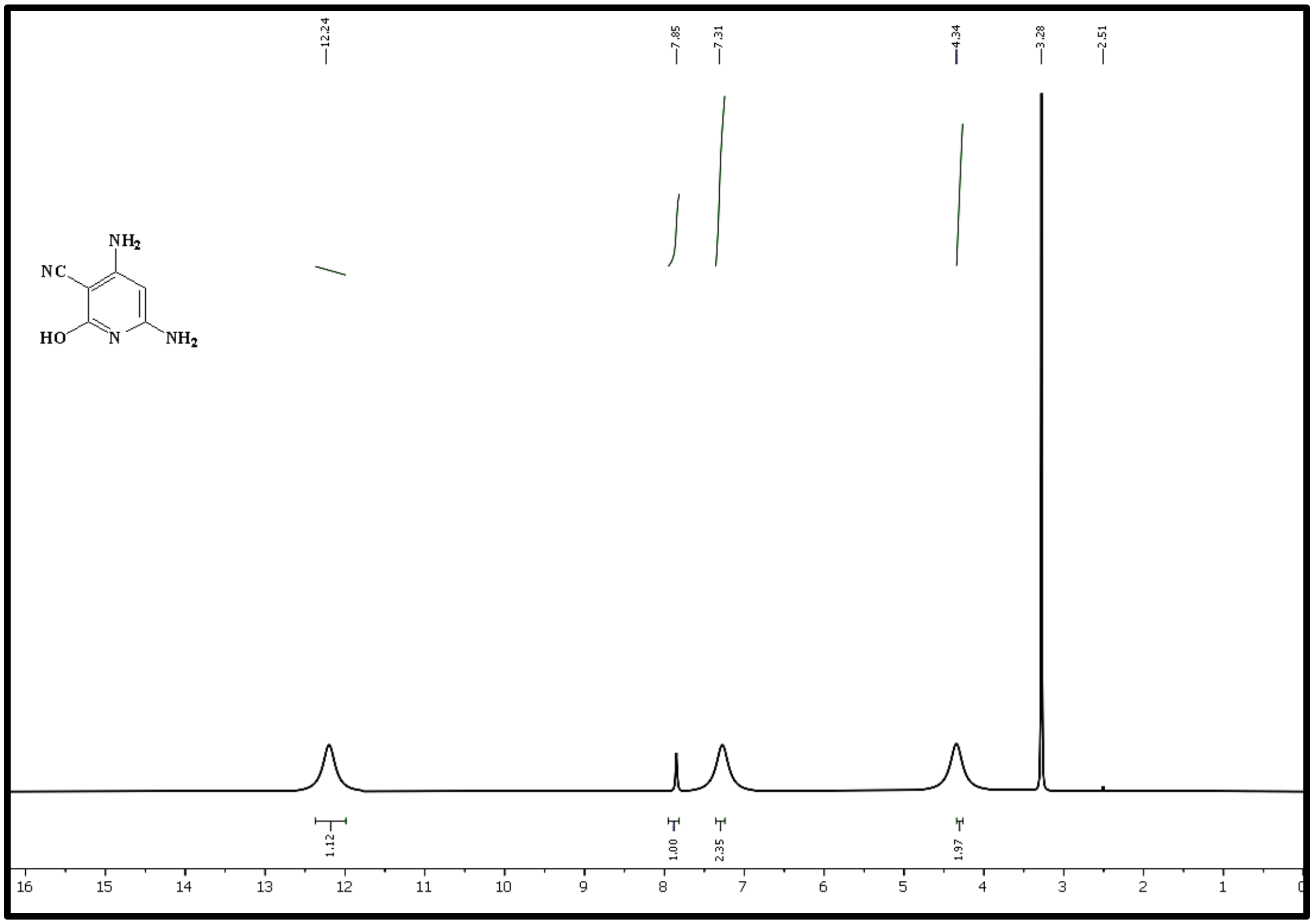

D_2O Spectrum of (7):

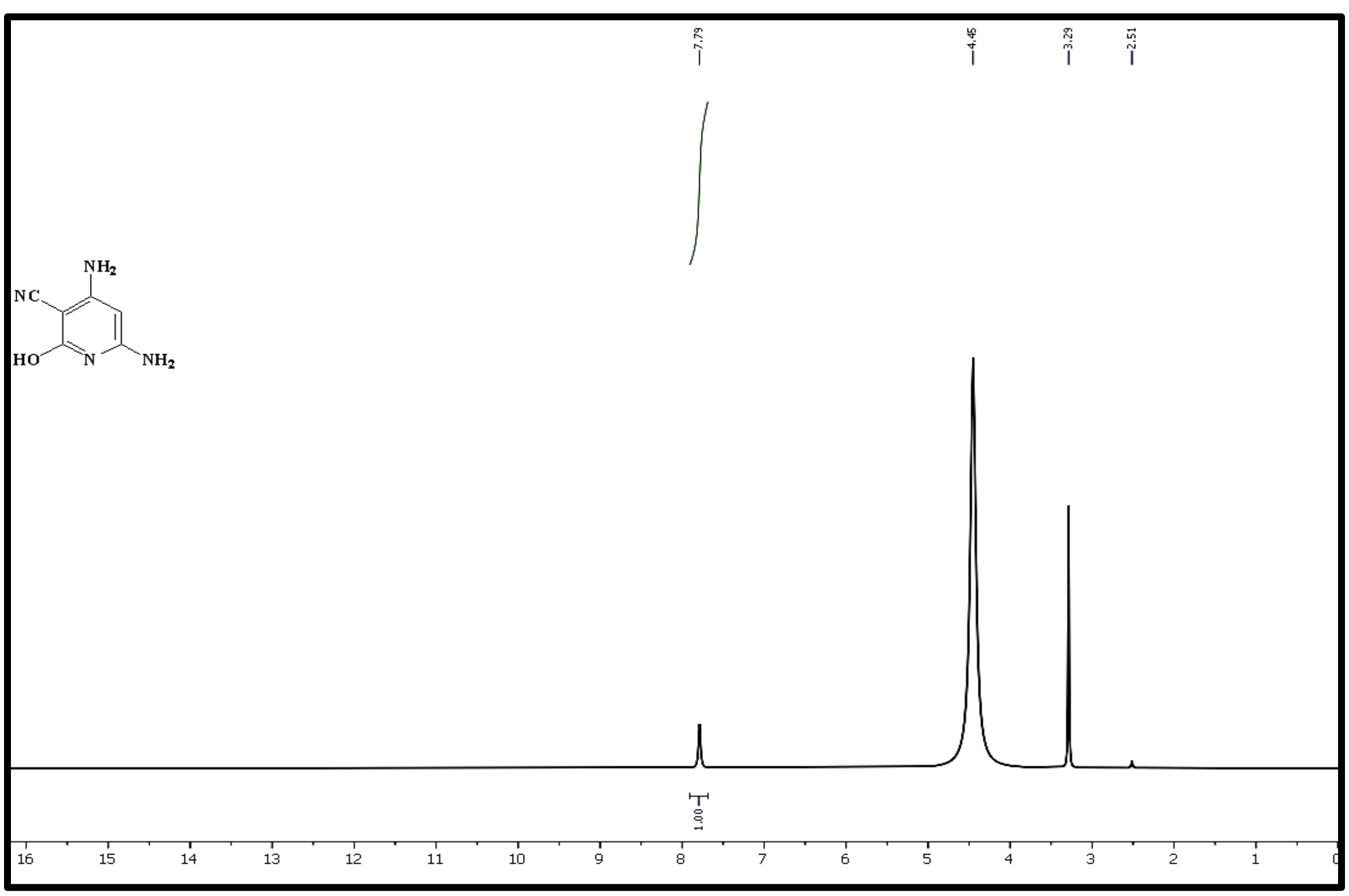

^{13}C-NMR Spectrum of (7):

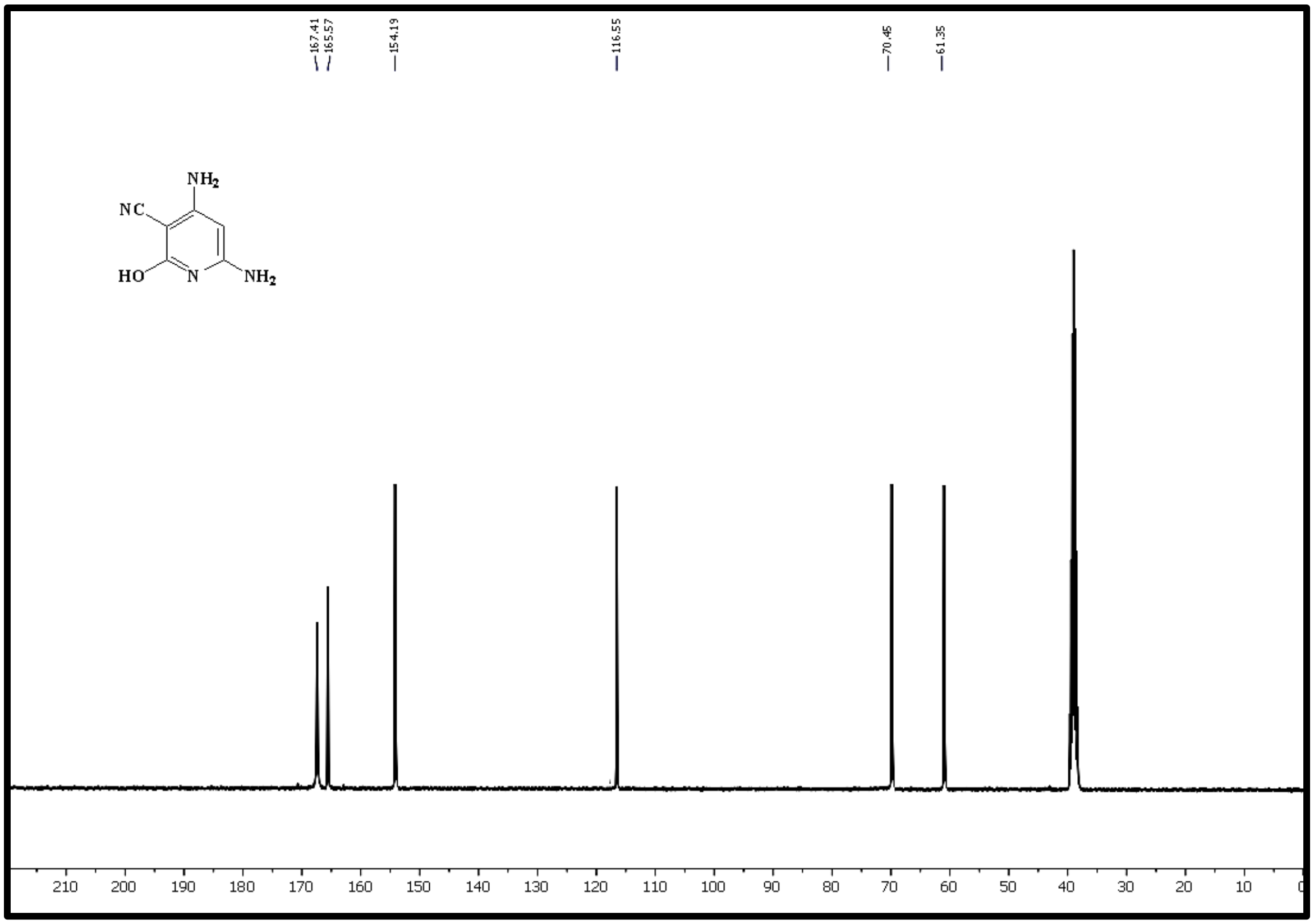

167.41
165.57
154.19
116.55
70.45
61.35
NH2
NC
HO
N
NH2
210 200 190 180 170 160 150 140 130 120 110 100 90 80 70 60 50 40 30 20 10 0

Environmental and Health Safety Guidelines for Quartz Nanoparticles

The utilization of quartz nanoparticles in heterogeneous catalysis requires careful consideration of potential occupational health and environmental safety hazards. Chronic inhalation of crystalline silica and quartz nanomaterials can induce pulmonary inflammation, oxidative stress via the generation of reactive oxygen species (ROS), and long-term fibrogenic effects. To mitigate these risks, comprehensive safety protocols must be integrated into laboratory and industrial workflows, including the mandatory use of certified particulate respirators (such as N95 or FFP3), protective nitrile gloves, and closed-system handling. Furthermore, all dry powder manipulations should be strictly restricted to certified chemical fume hoods to prevent aerosolization, and accidental spills must be carefully dampened with water prior to collection and disposal as hazardous nanoscale waste [1-3].

References

1. Fubini, B., & Areán, I. O. (1999). Chemical aspects of the toxicity of inhaled mineral dusts. *Chemical Society Reviews*, 28(6), 373-382.
2. Castranova, V. (2000). Generation of oxygen radicals and lung injury induced by coalmine dust and quartz. *Environmental Health Perspectives*, 108(Suppl 4), 675-679.
3. Donaldson, K., & Borm, P. J. (1998). The quartz hazard: a variable entity. *Annals of Occupational Hygiene*, 42(5), 287-294.
